# Acute Effects and Safety of Caffeine Ingestion in Adolescent Athletes: A Systematic Review and Three-Level Meta-Analysis

**DOI:** 10.3390/nu18152520

**Published:** 2026-08-03

**Authors:** Lantao Liu, Ming Chen, Hengzhi Deng, Hansen Li, Qiaoyun Wang

**Affiliations:** 1 Department of Physical Education, Central South University, Changsha 410083, China; csu_llt@csu.edu.cn; 2 School of Physical Education, Kunming University of Science and Technology, Kunming 650500, China; chenming061010@gmail.com; 3 Faculty of Sports and Exercise Science, University of Malaya, Kuala Lumpur 50603, Malaysia; denghengzhi66@gmail.com; 4 School of Physical Education, Sichuan Agricultural University, Ya’an 625014, China; hanson-swu@foxmail.com

**Keywords:** caffeine, adolescent athletes, ergogenic aid, three-level meta-analysis, physical performance, cluster-robust variance

## Abstract

**Background and Objectives:** Evidence on the acute ergogenic effects of caffeine in adolescent athletes remains limited. This systematic review quantified acute performance-related effects and summarised adverse-event reporting in this population. **Methods:** PubMed and Web of Science were searched from inception to 13 May 2026 and SPORTDiscus was additionally searched from inception to 15 July 2026 for placebo-controlled studies of acute, dose-defined caffeine ingestion in adolescent athletes aged 10–19 years. Paired Hedges’ g values were synthesised separately across six outcome domains using three-level random-effects models with CR2 cluster-robust inference. Risk of bias and certainty of evidence were assessed using RoB 2 and GRADE. Safety findings were summarised narratively. **Results:** Thirty-five studies were included, of which 32, representing a total of 726 participants, contributed 363 effect sizes. In the primary analysis, which excluded a priori a small number of extreme combat-sport count-test effects arising from implausibly small reported standard deviations (Hedges’ g > 3.0), caffeine produced a small favourable average effect on physical performance (g = 0.33, 95% CI 0.17 to 0.49). When all effect sizes were included in a sensitivity analysis, the estimate was larger but inflated by these extreme values (g = 0.43, 95% CI 0.20 to 0.67) and was accompanied by substantial heterogeneity (*I*^2^ = 84%), a prediction interval crossing the null (−0.71 to 1.58), and significant small-study asymmetry. Pooled effects were not statistically significant for sport-specific performance, perceptual response, or physiology. Cognitive findings were based on only two study clusters and were highly uncertain, while psychological outcomes were summarised narratively because the evidence was too sparse and heterogeneous for a meaningful pooled estimate. Exploratory moderator analyses did not provide reliable evidence that effects differed by caffeine dose or ingestion timing. No serious adverse events were reported, but adverse-event assessment was inconsistent. **Conclusions:** Acute caffeine ingestion may produce a small and uncertain benefit for selected physical-performance tasks in adolescent athletes. Adverse-event reporting was inconsistent, and current evidence is insufficient to support routine caffeine use in youth sport. These findings are specific to acute caffeine exposure under controlled experimental conditions in adolescent athletes and should not be extrapolated to children, non-athlete adolescents, repeated or chronic caffeine use, or energy-drink consumption.

## 1. Introduction

Caffeine (1,3,7-trimethylxanthine) is one of the most extensively studied and widely used ergogenic aids in sport. The International Society of Sports Nutrition concluded that caffeine acutely enhances many, though not all, aspects of exercise performance, with the strongest support for moderate doses of 3–6 mg·kg^−1^ taken approximately 60 min before exercise [1]. Domain-specific meta-analyses in predominantly adult samples have quantified these benefits: small-to-moderate improvements in maximal muscle strength and power [2], a small but consistent improvement in endurance performance [3], and a reliable reduction in the rating of perceived exertion during exercise [4]. However, the adult evidence does not imply a uniform ergogenic response. The same position stand reports wide variability in endurance outcomes and mixed evidence for sport skills and reactive agility, indicating that caffeine should be treated as a task-, dose-, and context-sensitive intervention rather than a general performance enhancer. Mechanistically, these effects are attributed primarily to the antagonism of central adenosine receptors, which lowers perceived exertion and pain and increases motor-unit recruitment and arousal, with additional peripheral effects on calcium handling and substrate use. This mechanism is relevant to adolescent sport because many competitive tasks depend not only on force production, but also on effort perception, pain tolerance, decision speed, attention, and repeated high-intensity actions. The same central stimulation may also produce unwanted responses in susceptible youth, including sleep problems, anxiety, faster heart rate, restlessness, and other symptoms. Thus, in adolescent athletes, efficacy and tolerability should be interpreted jointly rather than as separate questions [1].

Adolescence is commonly defined as the period from 10 to 19 years and is characterised by rapid physical, cognitive, and psychosocial growth. This developmental context limits direct extrapolation from adult sport nutrition studies. A comparable absolute caffeine dose can correspond to a larger body-mass-adjusted exposure in a lighter athlete, while maturation status, sleep need, anxiety susceptibility, habitual caffeine intake, training age, and genotype may modify both ergogenic and adverse responses [1]. Temple et al., [5] emphasised that paediatric caffeine guidance still rests largely on decades-old data collected in adults, and that although moderate intake has been considered relatively low risk in young people, higher doses can produce physiological, psychological, and behavioural harm, particularly in vulnerable subgroups. Adolescents differ from adults in body mass, caffeine pharmacokinetics and clearance, habitual intake, and maturation status, so the magnitude and even the direction of ergogenic and physiological responses cannot simply be extrapolated from adult data [1]. A quantitative, adolescent-specific evidence base is therefore both scientifically and practically important.

Findings from individual studies of adolescent athletes are inconsistent across tasks and settings, with benefits reported for some physical-performance outcomes but not for several sport-specific skills or related responses [6,7,8,9,10]. Differences in dose, ingestion timing, sport, participant characteristics, and study design further limit interpretation, supporting the need for an adolescent-specific quantitative synthesis.

To our knowledge, no previous systematic review has quantitatively synthesised the acute effects and safety of caffeine specifically in adolescent athletes. Existing syntheses have focused either on adults or on specific subgroups; for example, Mielgo-Ayuso et al., (2019) [11] compared caffeine responses between sexes. They have also typically used conventional random-effects models that treat the multiple effect sizes extracted from a single crossover trial as independent. Because a single adolescent trial commonly contributes many dependent effects across doses, ingestion times, sport-specific actions, jumps, sprints, and physiological markers, ignoring this dependence underestimates uncertainty and can overstate precision and statistical significance [12]. This review therefore estimated the acute effects of caffeine ingestion on exercise performance and related responses in adolescent athletes aged 10–19 years using three-level random-effects models with cluster-robust variance estimation, synthesised separately within six pre-specified outcome domains. The population comprised adolescent athletes; the intervention was acute, dose-defined caffeine before exercise; the comparator was placebo; and the outcomes were physical performance, sport-specific performance, physiology, perceptual response, psychological response, and cognitive performance.

## 2. Methods

### 2.1. Reporting Guideline and Protocol Registration

This systematic review and meta-analysis were conducted and reported in accordance with the PRISMA 2020 statement in Supplementary Table S1. The protocol was prospectively registered on the Open Science Framework (OSF; https://osf.io/t2ea3 (accessed on 1 June 2026); https://doi.org/10.17605/OSF.IO/PXAGY).

### 2.2. Eligibility Criteria

Eligibility was defined according to the PICOS framework.

Population. Eligible participants were adolescent athletes aged 10–19 years, inclusive (i.e., ≥10 and <20 years at the time of caffeine exposure), who were engaged in organised sport training or competition. Studies of children younger than 10 years, adults aged 20 years or older, and non-athletic adolescent populations were excluded. Studies containing participants outside the eligible age range were included only when data for athletes aged 10–19 years were reported separately or could be obtained from the study authors. When the full participant age range was not reported, eligibility was evaluated using the available numerical age data or an explicitly reported competition age category.

Intervention. Eligible interventions involved acute caffeine exposure before or during an exercise, sport, cognitive, perceptual, physiological, or safety assessment. Caffeine dose was extracted preferentially as mg·kg^−1^ body mass, and absolute dose (mg) was extracted when available. Administration form was coded as capsule, coffee, caffeinated beverage, energy drink, sports drink, chewing gum, mouth rinse, or other. Timing was coded as minutes before the main outcome test and, where relevant, as morning, afternoon, or evening ingestion. A study was eligible for quantitative synthesis only when the caffeine dose was defined and the intervention and comparator used the same delivery vehicle and were matched for all reported non-caffeine ingredients, such that caffeine was the intended between-condition difference. Capsules, coffee, caffeinated beverages, energy or sports drinks, chewing gum, and mouth rinse were not classified as isolating or non-isolating caffeine effects solely because of delivery form; eligibility depended on comparator matching. Multi-ingredient products with unmatched active co-ingredients, unclear comparator composition, or insufficient information to isolate caffeine were excluded from the quantitative synthesis and summarised narratively.

Comparator. Eligible comparators were placebo, decaffeinated control, caffeine-free beverage, low-caffeine condition, or usual condition. Comparator composition, blinding similarity, taste matching, and caffeine content were extracted, and comparator ambiguity was considered during risk-of-bias assessment.

Outcomes. The primary outcomes were acute exercise or sport performance and safety. Performance outcomes were classified as aerobic endurance, intermittent exercise capacity, sprint or acceleration, repeated-sprint ability, change in direction or agility, jump performance, power output, maximal strength, muscular endurance, sport-specific technical skill, and simulated match performance. Secondary outcomes were cognitive performance, perceptual response, psychological response, and physiological response. Cognitive outcomes included reaction time, attention, vigilance, decision-making, inhibitory control, and executive function. Perceptual outcomes included rating of perceived exertion, task-related pain, and fatigue. Psychological outcomes included mood, anxiety, arousal, and subjective vitality. Physiological outcomes included heart rate, blood pressure, blood lactate, blood glucose, ventilatory measures, catecholamines, and other reported markers. Safety outcomes included headache, gastrointestinal discomfort, nausea, diarrhoea, abdominal pain, anxiety, nervousness, palpitations, tachycardia, tremor, dizziness, sleep disturbance, insomnia, increased urination, and any serious adverse event.

Study design. Randomised crossover trials, randomised parallel-group trials, placebo-controlled trials, and controlled trials with extractable acute caffeine effects were eligible. Non-randomised studies were eligible only for narrative synthesis unless their design allowed for valid extraction of the intervention effect and an appropriate risk-of-bias tool was applied. Animal studies, in vitro studies, adult-only studies, chronic training interventions without separable acute caffeine effects, and studies without extractable outcome data were excluded.

### 2.3. Information Sources and Search Strategy

PubMed and Web of Science Core Collection were searched from database inception to 13 May 2026. Following peer-review feedback, SPORTDiscus via EBSCOhost was additionally searched from database inception to 15 July 2026 using the same eligibility criteria and screening procedures as the original searches. No date restrictions were applied; searches were restricted to English-language records. Reference lists of included studies and relevant reviews were screened manually. Records were exported to Zotero, and duplicates were removed before screening. Example PubMed search string: (caffeine[Title/Abstract] OR coffee[Title/Abstract] OR caffeinated beverage[Title/Abstract] OR energy drink[Title/Abstract] OR caffeine supplementation[Title/Abstract] OR caffeine ingestion[Title/Abstract]) AND (child[Title/Abstract] OR children[Title/Abstract] OR adolescent[Title/Abstract] OR adolescents[Title/Abstract] OR youth[Title/Abstract] OR teenager*[Title/Abstract] OR pediatric[Title/Abstract] OR paediatric [Title/Abstract]) AND (athlete*[Title/Abstract] OR trained[Title/Abstract] OR sport*[Title/Abstract] OR player*[Title/Abstract]) AND (acute[Title/Abstract] OR ingestion[Title/Abstract] OR performance[Title/Abstract] OR cognition[Title/Abstract] OR reaction time[Title/Abstract] OR perceived exertion[Title/Abstract] OR safety[Title/Abstract] OR adverse event*[Title/Abstract] OR side effect*[Title/Abstract] OR sleep[Title/Abstract] OR heart rate[Title/Abstract] OR blood pressure[Title/Abstract] OR anxiety[Title/Abstract] OR gastrointestinal[Title/Abstract]). Example Web of Science string: TS = ((caffeine OR coffee OR caffeinated beverage OR energy drink OR caffeine ingestion) AND (child* OR adolescent* OR youth OR teenager* OR pediatric OR paediatric) AND (athlete* OR trained OR sport* OR player*) AND (acute OR ingestion OR performance OR cognition OR perceived exertion OR safety OR adverse event* OR side effect* OR sleep OR heart rate OR blood pressure OR anxiety OR gastrointestinal)). The full strategy is provided in Supplementary Table S2.

### 2.4. Study Selection

All retrieved records were imported into Zotero and deduplicated manually by an independent reviewer (MC). The deduplicated records were then independently screened by two reviewers (M.C. and H.Z.D.) based on predefined inclusion and exclusion criteria. Records considered potentially relevant by either reviewer were retrieved for full-text assessment. The 2 independent reviewers reviewed the full texts of selected articles for final inclusion. Any disagreements during screening or full-text assessment were resolved through discussion; if consensus could not be reached, a third reviewer (Q.Y.W.) adjudicated the decision.

### 2.5. Data Extraction

Two reviewers independently extracted data using a piloted form. Extracted information included author, year, country, study design, sample size, sex, chronological age, body mass, height, BMI, sport, competitive level, training years, weekly training volume, pubertal stage, biological-maturity indicator, menstrual status (where applicable), habitual caffeine intake, diet control, sleep control, pre-test exercise control, caffeine dose (mg·kg^−1^ and absolute mg), administration form, ingestion timing, comparator composition, blinding method, washout duration, test protocol, outcome type and unit, means, standard deviations, change scores, paired correlations, adverse events, sleep outcomes, funding source, and conflicts of interest. For RPE, values were extracted at the end of the standardised exercise task or at the closest comparable endpoint. When multiple time points were reported, intermediate values during non-comparable exercise stages were not pooled unless they represented the same task phase across conditions. Variables not described in the original report were coded as “not reported,” and no assumptions were made about participant or study characteristics. For adverse events, “not reported” was distinguished from “assessed with no events reported” whenever the original article provided sufficient information. When data were available only in figures, they were extracted by two reviewers independently using WebPlotDigitizer.

### 2.6. Risk-of-Bias Assessment

Risk of bias in randomised parallel-group trials was assessed using RoB 2 (Supplementary Table S3), covering the randomisation process, deviations from intended interventions, missing outcome data, outcome measurement, and selection of the reported result. For randomised crossover trials, the RoB 2 crossover variant was applied, with additional consideration of crossover-specific issues such as carry-over and period effects. Non-randomised studies, if retained, were assessed using ROBINS-I. Two reviewers independently assessed risk of bias, and disagreements were resolved through discussion or consultation with a third reviewer. The PEDro scale (Supplementary Table S4) was completed as a supplementary assessment of methodological quality and was reported descriptively.

### 2.7. Effect-Size Calculation

For continuous outcomes, standardised mean differences were first calculated and then converted to Hedges’ g by applying the small-sample correction factor J. For crossover studies, the standardised mean difference was calculated following Morris and DeShon [13] using the pooled between-condition standard deviation. For parallel-group studies, the pooled post-intervention standard deviation was used, with change-score data used when appropriate. For performance, cognitive, perceptual, and psychological outcomes, positive values indicated a response favouring caffeine; time-based outcomes (e.g., sprint and agility time) were reverse-coded so that positive values indicated better performance. For physiological outcomes, the coding direction was pre-specified for interpretability and reported separately from performance interpretation, because changes such as higher heart rate or blood lactate are not inherently favourable or unfavourable. For adverse events, incidence data and descriptive safety information were extracted. No quantitative safety synthesis was undertaken because adverse-event definitions, ascertainment methods, and follow-up periods were not sufficiently comparable across studies. None of the included crossover studies reported a usable within-participant correlation. Therefore, r = 0.50 was imputed for variance estimation in the primary analysis, with r = 0.25 and r = 0.75 examined in sensitivity analyses. The imputed correlation affected the sampling variance, not the point estimate of Hedges’ g [12]. Effect-size calculations are described in Supplementary Note S1.

### 2.8. Quantitative Synthesis

Because most trials used crossover designs and reported multiple dependent effects from the same participants, primary analyses used three-level random-effects models with effect sizes nested within study clusters [14], estimating sampling error, within-study heterogeneity, and between-study heterogeneity by restricted maximum likelihood. Cluster-robust variance estimation with CR2 adjustment and Satterthwaite degrees of freedom was used for pooled estimates and moderator tests. Separate models were fitted for each pre-specified outcome domain; domains were not collapsed into a single omnibus effect. A minimum of two independent study clusters was required for model estimation. However, a pooled estimate was not presented when the available evidence was too sparse or structurally unbalanced to support a meaningful domain-level summary. Psychological outcomes were therefore synthesised narratively because only two independent clusters contributed data, 20 of the 26 effect sizes came from one cluster, and the outcomes showed substantial between-effect heterogeneity. Heterogeneity was reported using Q, *I*^2^, τ^2^, and 95% prediction intervals where estimable, with prediction intervals interpreted cautiously in sparse domains. Statistical significance was defined as two-sided *p* < 0.05, but interpretation emphasised effect size, confidence intervals and prediction intervals, heterogeneity, risk of bias, and GRADE certainty rather than *p*-values alone. For crossover trials, paired effect sizes and their variances were computed using only first-period-comparable, within-participant difference data following Morris and DeShon (2002) [13]; where period or sequence effects could not be excluded from the original report, the corresponding study was flagged in the risk-of-bias assessment and examined in sensitivity analysis. The imputed within-participant correlation (0.50, with sensitivity values of 0.25 and 0.75) entered both the effect-size variance and the covariance among dependent effects within a cluster. All analyses were conducted in R (version 4.5.3) using the metafor package (version 5.0-1) for the three-level random-effects models and the clubSandwich package (version 0.6.2) for CR2 cluster-robust variance estimation with Satterthwaite degrees of freedom.

### 2.9. Moderator and Subgroup Analyses

Pre-specified moderator analyses were conducted when enough independent study clusters were available. Candidate moderators were chronological age, maturity stage, sex, sport category, competitive level, caffeine dose, administration form, ingestion timing, habitual caffeine intake, test setting, and study design. Chronological age was examined continuously and, where sufficient independent study clusters were available, categorically as early adolescence (10–14 years) and late adolescence (15–19 years), consistent with the review’s pre-specified 10–19-year eligibility criterion. Maturity was analysed as pre-pubertal, mid-pubertal, and post-pubertal, and the sport category as endurance, power or strength, team sport, racket sport, combat sport, and skill-dominant sport. Dose was analysed both continuously (mg·kg^−1^) and categorically as low (<4 mg·kg^−1^), moderate (4–6 mg·kg^−1^), and high (>6 mg·kg^−1^). All moderator analyses were considered exploratory because dose, sport, sex, timing, administration form, and study design were not independently distributed across studies [15,16].

### 2.10. Publication Bias and Sensitivity Analyses

Funnel plots were produced only for domains with enough effect sizes for descriptive inspection. Formal Egger or Begg tests were interpreted only when at least 10 independent study clusters were available, because effect-size dependence and sparse clusters can destabilise asymmetry tests. To avoid overstating precision from dependent effects, the Egger-type regression test of funnel asymmetry was fitted as a multilevel (three-level) extension with cluster-robust (CR2) standard errors rather than on the pooled effect sizes treated as independent, and the result was interpreted as a descriptive diagnostic of small-study effects. Sensitivity analyses included varying the assumed paired correlation; leave-one-cluster-out analysis; and excluding high-risk-of-bias studies, studies with undefined dose, multi-ingredient energy- or sports-drink studies, adult mixed samples, studies without systematic adverse-event assessment, and extreme effect sizes defined a priori as Hedges’ g > 3.0 [17]. For the physical-performance domain, in which these extreme effects were concentrated, the a priori exclusion of effect sizes with Hedges’ g > 3.0 was adopted as the primary analysis, with the model including all effect sizes reported as a sensitivity analysis.

### 2.11. Certainty of Evidence

Certainty of evidence was assessed using GRADE for each main outcome domain by considering risk of bias, inconsistency, indirectness, imprecision, and publication bias. Indirectness was judged with particular attention to whether participants were adolescent athletes, whether maturity and training level were reported, and whether adult-athlete evidence was being used only as background rather than as direct evidence. GRADE assessments were completed by 1 reviewer (MC) and reviewed by a second (LTL).

## 3. Results

### 3.1. Literature Screening

**Figure 1:**
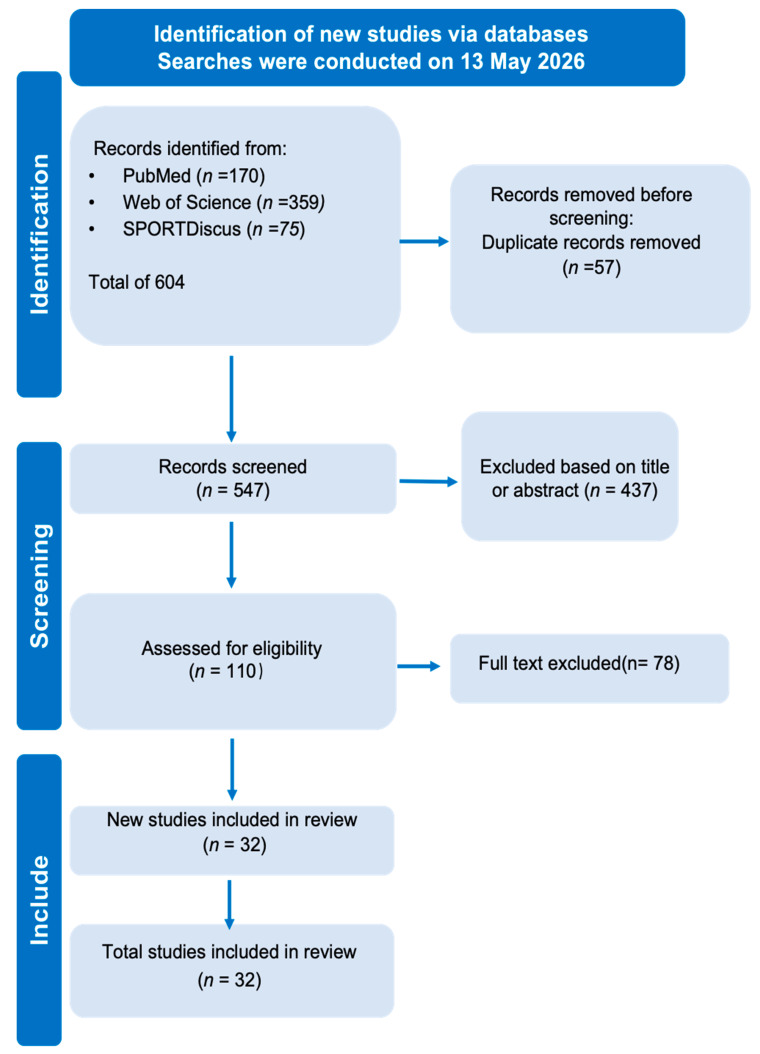
PRISMA 2020 flow diagram of the literature search and screening.

The combined search retrieved 604 records (170 from PubMed, 359 from Web of Science, and 75 from SPORTDiscus). After removing 57 duplicates, 547 records were screened, 437 were excluded by title and abstract, and 110 full texts were assessed for eligibility, of which 75 were excluded. Thirty-five studies were included in the review; 32 were included in the quantitative synthesis (Figure 1, PRISMA flow diagram).

### 3.2. Characteristics of Included Studies

The quantitative synthesis comprised 32 studies, representing a total of 726 participants and contributing 363 effect sizes across six outcome domains (Supplementary Table S5) [6,7,8,9,10,16,18,19,20,21,22,23,24,25,26,27,28,29,30,31,32,33,34,35,36,37,38,39,40,41,42,43]. Most reports used randomised, double-blind, placebo-controlled crossover designs. A single randomised trial, reported in two companion publications involving the same 54 elite youth soccer players, used a balanced-placebo independent-groups design [18,28]. The sports included soccer, taekwondo, basketball, karate, tennis, handball, volleyball, judo, and cycling, among others. Most samples comprised trained or elite athletes, with only one recreational sample. Caffeine doses ranged from 1 to 9 mg·kg^−1^ (most commonly 3–6 mg·kg^−1^), ingested 20–60 min before exercise. Samples were predominantly male; six clusters contributed female-only data, and a minority were mixed-sex. The effect sizes comprised 193 physical-performance, 50 sport-specific, 34 physiological, 45 perceptual, 26 psychological, and 15 cognitive outcomes. Detailed study- and participant-level characteristics, including age, sex, sport, competitive level, habitual caffeine intake, pubertal or biological maturity, and menstrual status where applicable, are presented in Supplementary Table S5 and Supplementary Table S5A. Reporting of key participant covariates was incomplete: biological or pubertal maturities were not reported in 28 of 32 reports, habitual caffeine intake was not reported in 7 of 32 reports, and menstrual status was not reported in 3 of the 9 reports that included female participants (Supplementary Table S5). Systematic adverse-event assessment was similarly inconsistent. These reporting gaps contributed to the GRADE assessment of indirectness. Across the included reports, study-level mean or median ages ranged from 14.07 to 18.3 years. Where minimum–maximum ages or explicit eligibility ranges were available, reported ages extended from approximately 12 to 19 years. No study clearly reported enrolling athletes aged 10–11 years, and several reports did not provide a complete participant age range.

### 3.3. Risk of Bias and Methodological Quality

RoB 2 judgements were predominantly “some concerns,” most often arising from incomplete reporting of the randomisation and allocation process and from limited information on blinding integrity and washout; no study was judged at high overall risk and none at low risk across all domains (Supplementary Table S3). The pervasive “some concerns” rating contributed to GRADE downgrading for risk of bias.

### 3.4. Main Analysis

**Figure 2:**
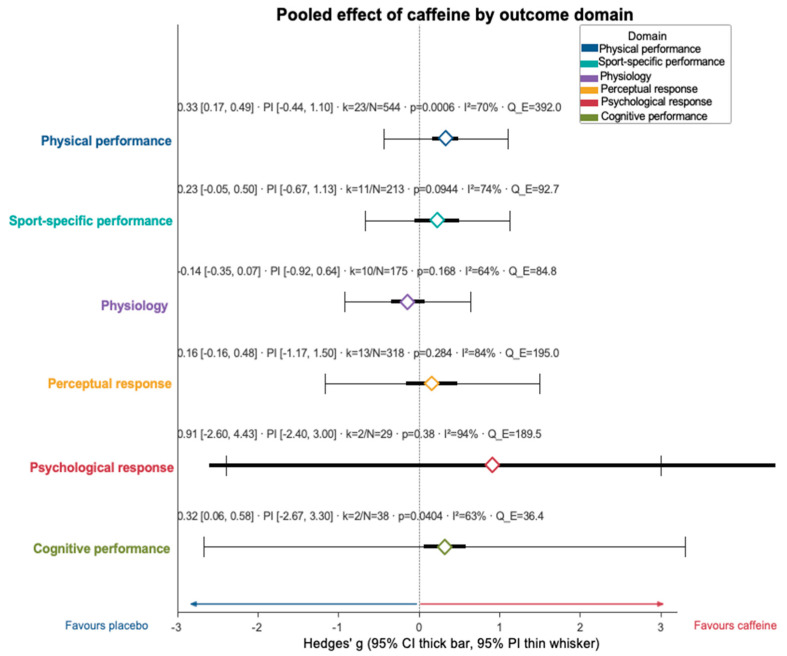
Orchard plot of pooled main effects by outcome domain. White diamonds indicate the three-level pooled Hedges’ g; thick black bars are CR2 cluster-robust 95% confidence intervals and thin capped whiskers are 95% prediction intervals. The dashed line marks zero effect; positive (right) favours caffeine. Each row is labelled with k studies, N, *p*, and *I*^2^.

**Figure 3:**
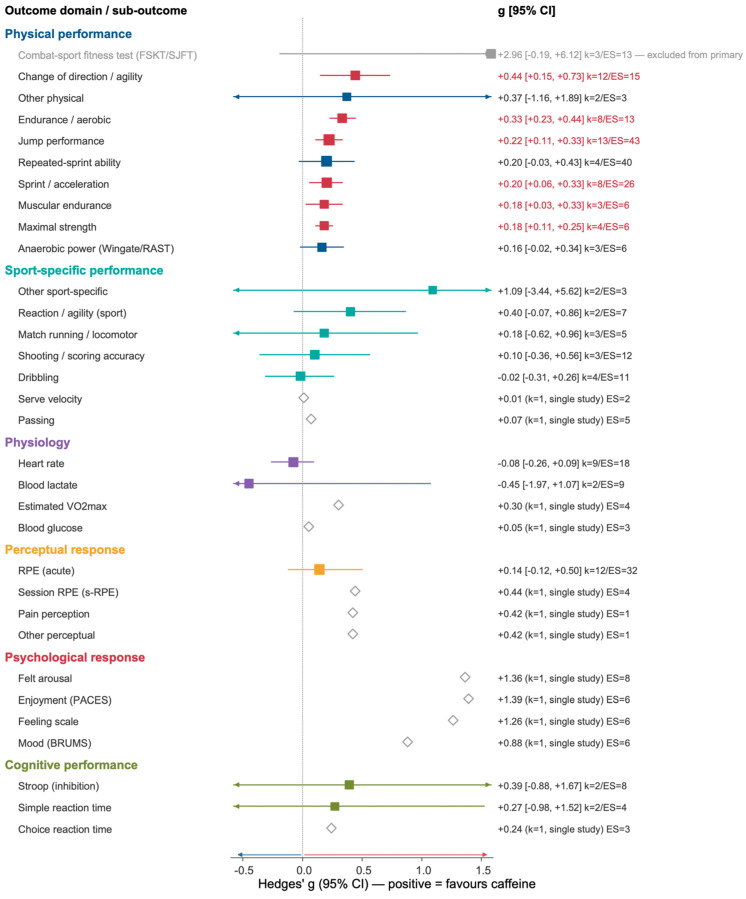
Synthetic forest plot of sub-outcomes within each domain. Filled squares are pooled estimates (k ≥ 2 studies; red = *p* < 0.05), sized by precision; hollow grey diamonds are precision-weighted means for single-study (k = 1) sub-outcomes (no CI, indicative only). Arrows mark CIs exceeding the axis. k and 95% CI are labelled for each estimate.

Orchard plots of the pooled main effects for each domain are shown in Figure 2, and the sub-outcome composite in Figure 3.

Compared with the placebo, caffeine produced a small favourable effect on overall physical performance. In the primary analysis, which excluded a priori the extreme combat-sport count-test effects with implausibly small standard deviations (Hedges’ g > 3.0; Section 2.10); the pooled estimate was g = 0.33 (95% CI 0.17 to 0.49; k = 189 effects, 23 studies). Including all effect sizes in a sensitivity analysis produced a larger but inflated estimate (k = 193 effects; g = 0.43, 95% CI 0.20 to 0.67; PI −0.71 to 1.58; CR2 *p* = 0.001; *I*^2^ = 84%). By contrast, the pooled effects for sport-specific performance (k = 50 effects, 11 studies; g = 0.23, 95% CI −0.05 to 0.50; PI −0.67 to 1.13; CR2 *p* = 0.094; *I*^2^ = 74%) and perceptual response (k = 45 effects, 13 studies; g = 0.16, 95% CI −0.16 to 0.48; PI −1.17 to 1.50; CR2 *p* = 0.284; *I*^2^ = 84%) did not reach significance, and the physiology domain showed a small, non-significant tendency in the unfavourable direction (k = 34 effects, 10 studies; g = −0.14, 95% CI −0.35 to 0.07; PI −0.92 to 0.64; CR2 *p* = 0.168; *I*^2^ = 64%), reflecting caffeine’s tendency to raise heart rate and blood lactate. Two domains were informed by only two study clusters. Cognitive performance (k = 15 effects, two studies; g = 0.32, 95% CI 0.06 to 0.58; PI −2.67 to 3.30; CR2 *p* = 0.040; *I*^2^ = 63%) reached nominal significance but should be regarded as preliminary. For psychological response, a pooled estimate is not reported: although 26 effect sizes were available, 20 came from a single study cluster and the outcomes were too heterogeneous (*I*^2^ = 94%) for a meaningful domain-level estimate, so this domain was summarised narratively (Section 3.5.3).

Within physical performance, the overall estimate was driven by consistent, lower-heterogeneity sub-outcome effects (Figure 3). Significant favourable pooled effects were observed for change-of-direction/agility (g = 0.44, 95% CI 0.15 to 0.73), aerobic endurance (g = 0.33, 95% CI 0.23 to 0.44; *I*^2^ = 0%), jump performance (g = 0.22, 95% CI 0.11 to 0.33; *I*^2^ = 18%), sprint/acceleration (g = 0.20, 95% CI 0.06 to 0.33), maximal strength (g = 0.18, 95% CI 0.11 to 0.25; *I*^2^ = 0%), and muscular endurance (g = 0.18, 95% CI 0.03 to 0.33). Repeated-sprint ability (g = 0.20, 95% CI −0.03 to 0.43) and anaerobic power (g = 0.16, 95% CI −0.02 to 0.34) were positive but non-significant. The combat-sport fitness test sub-outcome (FSKT/SJFT) produced an extreme estimate (g = 2.96, 95% CI −0.19 to 6.12; *I*^2^ = 94%) arising from a few small-sample taekwondo trials with very small standard deviations; it was retained separately and excluded from the narrative interpretation of physical performance to avoid inflating the domain effect. Sub-outcome decompositions for the other domains are shown in Figure 3.

### 3.5. Moderator Analyses

**Figure 4:**
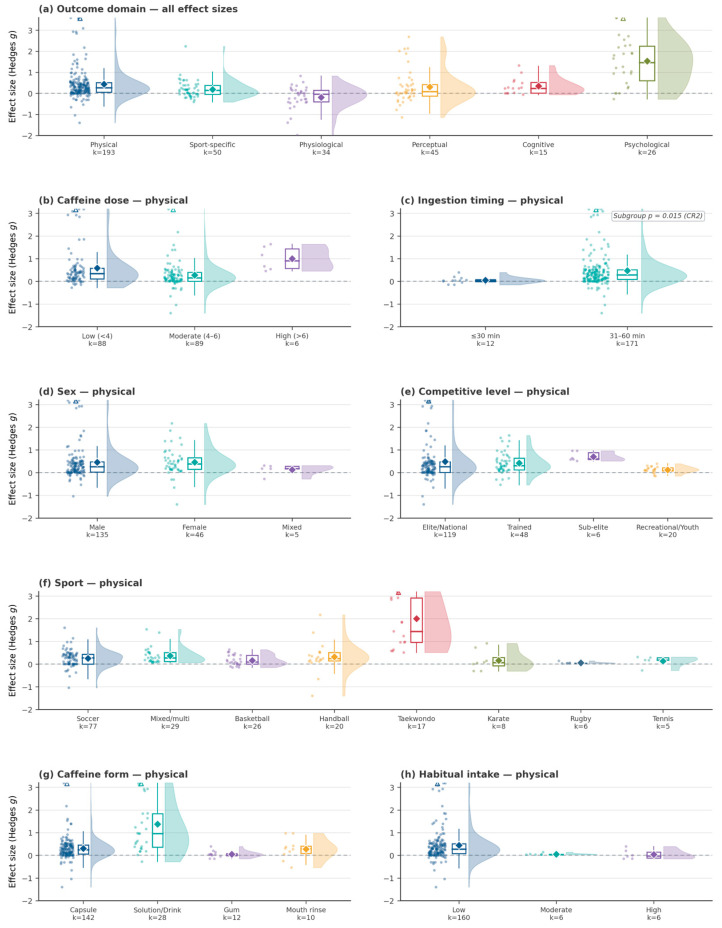
Subgroup rainclouds of the caffeine effect. Distribution of effect sizes (Hedges’ g; positive = favours caffeine) shown as jittered points (rain), a boxplot (median and IQR), and a half-violin density (cloud); the diamond marks the subgroup mean and the dashed line indicates no effect. Panel (**a**) covers all 363 effect sizes by outcome domain; panels (**b**–**h**) are restricted to the physical-performance domain (k = 193) and show caffeine dose, ingestion timing, sex, competitive level, sport, caffeine form, and habitual intake. k = number of effect sizes; levels with fewer than three effect sizes were omitted. Subgroup means are descriptive; the only inferential moderator test displayed is the pre-specified ingestion-timing contrast (*p* = 0.015). Open triangles mark effect sizes plotted above the axis (combat-sport, very small SD).

**Figure 5:**
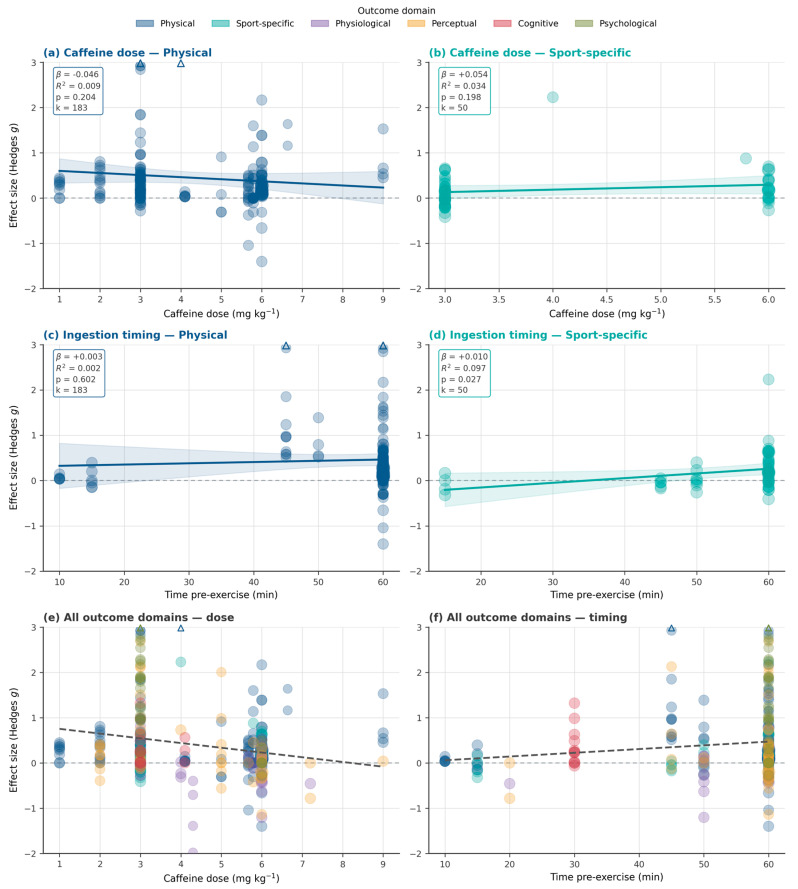
Dose–response and ingestion-timing visualisation (regression + bubble). Effect size (Hedges’ g) versus caffeine dose (panels (**a**,**b**,**e**)) and time pre-exercise (panels (**c**,**d**,**f**)) for physical and sport-specific performance and, pooled, for all outcome domains. Points are scaled so that bubble area is proportional to precision (1/SE); lines are ordinary-least-squares fits with 95% confidence bands and are for visualisation only. Open triangles mark effect sizes above the axis.

All moderator analyses were exploratory because caffeine dose, ingestion timing, sport, sex, competitive level, administration form, and study design were unevenly distributed and partly confounded across the included studies. No moderator provided sufficiently robust evidence of effect modification. The only relatively consistent pattern was observed for ingestion timing within the physical-performance domain, but this finding was based on an unbalanced comparison and should be considered hypothesis-generating. In contrast, no clear dose–response relationship emerged. The subgroup distributions are presented descriptively in Figure 4, whereas Figure 5 illustrates the continuous associations with dose and ingestion timing. These visualisations should not be interpreted as confirmatory evidence of moderation.

#### 3.5.1. Physical Performance

Within physical performance (k = 193), ingestion timing was the only moderator with a consistent signal. Effects for caffeine taken 31–60 min before exercise (g = 0.50, 95% CI 0.21 to 0.78, 21 clusters) were larger than for ingestion ≤ 30 min before (g = 0.11, 95% CI −0.10 to 0.33, 3 clusters; between-group *p* = 0.015), consistent with the time course of caffeine absorption (Figure 4c; Figure 5c). However, because the ≤30 min category contained only three independent clusters and timing was partly confounded with dose, sport, administration form, and study design, this contrast was considered statistically unstable and insufficient to establish effect modification by ingestion timing. The dose-category test was also significant (between-group *p* = 0.001) but did not describe a monotonic dose–response: the point estimates were g = 0.43 (low, <4 mg·kg^−1^), g = 0.26 (moderate, 4–6 mg·kg^−1^), and g = 0.82 (high, >6 mg·kg^−1^, only two clusters with a very wide CI). This pattern conflicted with the non-significant continuous dose slope (β = 0.052, SE = 0.024, CR2 *p* = 0.164) and therefore reflects unbalanced categorisation chiefly the sparse, imprecise high-dose stratum rather than a true dose effect (Figure 4b; Figure 5a,e). Effects did not differ significantly by sex (male g = 0.55, female g = 0.44; between-group *p* = 0.170), competitive level (between-group *p* = 0.530), or habitual intake (between-group *p* = 0.180). The widest and most right-shifted distributions occurred in combat sports, particularly the taekwondo fitness-count effects, consistent with the small-standard-deviation inflation identified in the sensitivity analysis, whereas team-sport and endurance effects were smaller and more symmetric (Figure 4f). These extreme values are shown but should not be read as a genuinely larger combat-sport benefit.

#### 3.5.2. Sport-Specific Performance

No clear moderator pattern was observed for sport-specific performance. Differences by sex (*p* = 0.505), dose category (*p* = 0.086), and habitual caffeine intake (*p* = 0.110) were not statistically significant. The continuous dose slope was also non-significant (β = 0.097, SE = 0.059, CR2 *p* = 0.143). Thus, the available evidence does not indicate that caffeine dose or the assessed participant characteristics reliably modify sport-specific performance effects. At the sub-outcome level, reaction and agility tasks produced the largest estimate (g = 0.40, 95% CI −0.07 to 0.86; two clusters), whereas the estimates for shooting or scoring accuracy (g = 0.10) and dribbling (g = −0.02) were negligible. These comparisons were based on sparse evidence and should be interpreted descriptively. At the overall domain level, caffeine did not produce a clear improvement in sport-specific performance (g = 0.23, 95% CI −0.05 to 0.50).

#### 3.5.3. Other Domains

Within physiology, heart rate (g = −0.08, 9 clusters) and blood lactate (g = −0.45, 2 clusters) showed non-significant tendencies toward higher values with caffeine. Within the perceptual domain, acute RPE (g = 0.14, 12 clusters) showed no reliable change. In cognitive performance, Stroop interference (g = 0.39) and simple reaction time (g = 0.27) were positive but non-significant and limited to two clusters. All psychological-response sub-outcomes were derived from a single study cluster and were summarised descriptively, which explains the few extreme values dominating that domain in Figure 4a. The tendencies toward higher heart rate and blood lactate should not be interpreted as a favourable adaptation; they may reflect acute physiological stimulation, altered exercise output, or a possible safety signal depending on context and accompanying symptoms.

Viewed across domains (Figure 4a), physical performance and the performance-adjacent domains were centred just above zero, physiology straddled zero, and the psychological-response domain was dominated by a few extreme combat-sport values, a descriptive summary that mirrors the pooled estimates above.

### 3.6. Sensitivity Analysis and Precision

**Figure 6:**
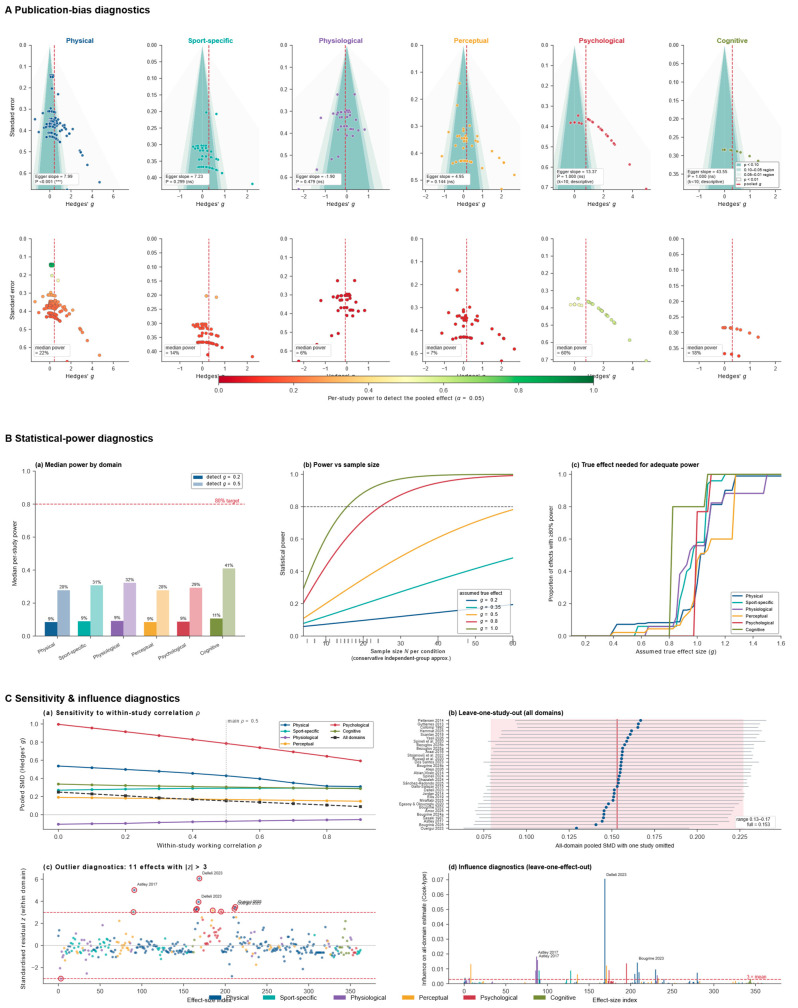
Publication bias, statistical power, and sensitivity analyses across the six outcome domains. (**A**) Funnel plots for small-study effects: contour-enhanced funnels (top row), showing each effect size against its standard error with significance contours and the domain pooled estimate marked, and power-enhanced “sunset” funnels (bottom row) colour-coded by each effect’s power to detect the pooled effect (green = adequate, red = underpowered). (**B**) Statistical-power diagnostics by domain: median per-study power for a small (g = 0.2) and a moderate (g = 0.5) effect, power versus per-condition sample size, and the proportion of effects reaching ≥80% power. (**C**) Sensitivity and influence analyses: robustness of the pooled estimate to the imputed within-study correlation ρ (**a**); leave-one-study-out re-estimation (**b**); outlier diagnostics flagging within-domain standardised residuals |z| > 3 (**c**); and leave-one-effect-out (Cook-type) influence (**d**). Effect sizes are Hedges’ g; colours denote the six outcome domains throughout. Exact slopes, standard errors, *p*-values, and power thresholds are reported in Section 3.6 and Supplementary Table S7 [6,7,8,9,10,16,18,19,20,21,22,23,24,25,26,27,28,29,30,31,32,33,34,35,36,37,38,39,40,41,42,43].

Publication bias was screened with Egger-type regression of each effect size on its standard error, fitted within the same correlated-and-hierarchical-effects (CHE) model with cluster-robust (CR2) variance estimation, so that the test of small-study effects respected the dependence among effect sizes. Among the four outcome domains with at least ten independent clusters, asymmetry was significant only for physical performance (slope = 7.99, SE = 1.99, *p* < 0.001): smaller trials reported systematically larger caffeine benefits. The all-domain diagnostic was likewise asymmetric (slope = 8.17, *p* = 0.015), whereas sport-specific performance (slope = 7.23, *p* = 0.299), perceptual responses (slope = 4.95, *p* = 0.144) and physiological responses (slope = −1.90, *p* = 0.479) showed no significant asymmetry. Cognitive and psychological responses each rested on only two clusters and could not be evaluated; their funnels are shown for completeness but interpreted descriptively (Figure 6A; Supplementary Table S7). Given the small number of studies per domain, the dependence among effect sizes, and the predominance of small studies, the power of the asymmetry test was limited, and the significant slopes should be read as suggestive rather than confirmatory; PET-PEESE correction was therefore not performed.

The statistical-power diagnostics were sobering. Under a conservative independent-group approximation (α = 0.05), the median per-study power to detect a small effect (g = 0.2) was only about 8–11% across domains, and the median power to detect a moderate effect (g = 0.5) reached only about 28–41%; no individual effect size attained 80% power (Figure 6B). Even against each domain’s own pooled estimate, median per-study power was modest: approximately 22% for physical performance and ≤14% for sport-specific performance, physiological responses, and perceptual responses (Figure 6A, lower row), indicating that the primary trials were systematically underpowered for the effects observed.

Sensitivity analyses indicated that the main-effect estimates were robust in direction. As the imputed within-study working correlation was varied from ρ = 0.0 to 0.9 (main analysis ρ = 0.5), the all-domain pooled estimate remained positive but declined modestly from g = 0.153 (ρ = 0.5) to 0.122 (ρ = 0.7) and 0.090 (ρ = 0.9), losing statistical significance only at the highest correlation (95% CI −0.019 to 0.199 at ρ = 0.9), while the physical-performance estimate was more stable (g = 0.429, 0.351 and 0.309 across the same ρ values) and remained significant throughout (Figure 6C(a)). Leave-one-study-out analyses confirmed this stability: the all-domain estimate ranged only from 0.13 to 0.17 with every removal, and the physical-performance estimate from 0.29 to 0.46, remaining positive and significant in every iteration (Figure 6C(b)); removal of Delleli et al. (2023) [32] was the most influential because of its large combat-sport effects, a pattern corroborated by the leave-one-effect-out influence (Cook-type) diagnostics (Figure 6C(d)). Outlier screening identified 11 effect sizes with a within-domain standardised residual |*z*| > 3, almost all of them extreme combat-sport kick-test outcomes ([24,26,32]; Figure 6C(c)). Removing these left the all-domain estimate essentially unchanged at *g* = 0.165 (95% CI 0.087 to 0.242), and removing all extreme effects (|*g*| > 3) attenuated the physical-performance estimate from 0.429 to 0.328 (95% CI 0.166 to 0.489) without altering its direction or significance. Taken together with the extreme combat-sport effects, the small-study asymmetry suggests that the physical-performance estimate may modestly overstate the true effect. These extreme effects arise from implausibly small reported standard deviations rather than from a genuinely larger combat-sport benefit: for the taekwondo maximal kick-count tests, the reported dispersion corresponds to a coefficient of variation below 1% (e.g., 141 ± 1.3 and 130 ± 1.0 kicks across 16 athletes), and within a single study [32], the same test is reported with standard deviations of 1–2 kicks in some subgroups but 6–10 kicks in others, an internal inconsistency that alone shifts the standardised effect from g ≈ 0.5 to g ≈ 3.0. We verified these values against the original reports; the anomaly therefore reflects the dispersion reported in the source studies rather than an extraction error. Because such near-zero denominators mechanically inflate the standardised mean difference, these extreme effects were excluded a priori (Hedges’ g > 3.0; Section 2.10) from the primary physical-performance analysis which yielded g = 0.33 (95% CI 0.17 to 0.49), whereas the model including all effect sizes (g = 0.43) is reported as a sensitivity analysis.

### 3.7. Certainty of Evidence

GRADE certainty (Table 1) was low for physical performance; this domain was downgraded for risk of bias, inconsistency, and small-study effects. Certainty was very low for sport-specific performance, physiology, perceptual response, psychological response, and cognitive performance owing to imprecision, inconsistency, indirectness, and, for the psychological and cognitive domains, reliance on only two study clusters.

### 3.8. Safety and Adverse Events

Safety findings were summarised narratively because adverse-event data were not sufficiently comparable for quantitative synthesis or formal GRADE assessment. Safety reporting was incomplete. No included study reported a serious adverse event, but this should not be interpreted as evidence that caffeine is safe for adolescent athletes. Safety monitoring was inconsistent across studies: of the 32 studies included in the quantitative synthesis, 10 actively assessed adverse events with a dedicated side-effect instrument, 11 only passively mentioned symptoms or physiological signals, and 11 did not assess adverse events at all. The absence of reported events in studies that did not actively assess them therefore reflects incomplete monitoring rather than demonstrated safety, and these three categories should be interpreted separately. Reported symptoms included headache, gastrointestinal symptoms, sleep disturbance or insomnia, tachycardia or palpitations, anxiety or nervousness, and increased urination (Supplementary Table S6).

**Figure 7:**
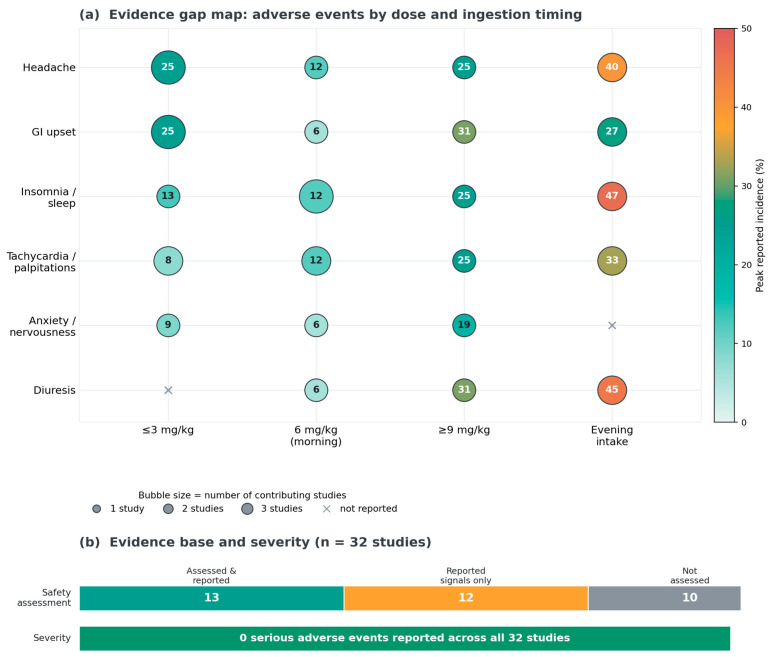
Evidence map of caffeine safety in adolescent athletes. (**a**) Evidence gap map of the six reported adverse-event categories (rows) against caffeine dose and ingestion timing (columns); bubble area is proportional to the number of contributing studies, colour encodes the peak reported incidence (%), the value is printed in each bubble, and a cross marks conditions for which the event was not reported. Incidence rises with high doses (≥9 mg·kg^−1^) and, most clearly, with evening ingestion. (**b**) Evidence base and severity across the 35 included studies: 13 actively assessed and reported adverse events with a dedicated instrument, 12 only passively mentioned symptoms or physiological signals, and 10 did not assess safety at all, while no serious adverse event was reported in any study. The absence of reported events in the latter two groups reflects incomplete monitoring rather than demonstrated safety.

Higher doses and evening ingestion were associated with more frequent symptoms. In the included youth studies, 9 mg·kg^−1^ was associated with more gastrointestinal, sleep, headache, and palpitation symptoms, and evening ingestion with more insomnia, headache, tachycardia, and diuresis. The defensible interpretation is therefore that these short-term trials did not report serious adverse events, but that adverse-event monitoring was incomplete, follow-up was short, and the evidence is insufficient to establish the safety of caffeine across adolescent athletes aged 10–19 years. These safety patterns are summarized in Figure 7.

## 4. Discussion

### 4.1. Principal Findings

This systematic review and three-level meta-analysis found that acute caffeine ingestion may provide a small benefit for selected physical-performance tasks in adolescent athletes. The clearest support was for general physical-performance outcomes, while sport-specific performance, perceptual response, and physiological response did not show clear pooled differences. Cognitive and psychological outcomes were based on very limited evidence. Overall, the findings suggest that caffeine may help some physical tasks, but they do not support routine caffeine use in youth sport.

These findings must be interpreted in light of the limitations of the youth-specific evidence base and the safety concerns surrounding caffeine use in adolescents. Previous reviews have shown that evidence specific to young people is much more limited than evidence in adults, especially regarding exercise performance, repeated caffeine use, and long-term safety [5,44]. The present review extends this literature by quantitatively identifying a small favourable effect on selected physical-performance outcomes in adolescent athletes. However, the low certainty, wide prediction interval and small-study effects observed here reinforce, rather than resolve, the caution expressed in previous youth-focused reviews [45,46].

### 4.2. Moderator Analyses

Because caffeine dose, sport, sex, competitive level, ingestion timing, and study design were partly confounded across the 32 study clusters, all moderator analyses were considered exploratory [47]. No moderator provided sufficiently robust evidence of effect modification. A nominal difference was observed between the 31–60 min and ≤30 min timing categories, but the latter contained only three independent study clusters. Given this sparsity and the confounding of timing with dose, sport, administration form, and study design, the finding is too unstable to identify an optimal ingestion time or support a practical timing recommendation [1]. The dose findings did not support the “higher is better” interpretation. The apparent high-dose advantage was based on sparse evidence and was not supported by a clear continuous dose–response pattern. This is important because adult position statements usually describe moderate doses as effective, while also noting that higher doses may increase side effects without providing extra performance benefits. The present adolescent-athlete evidence is broadly consistent with that view. It does not show that higher doses are necessary, and it gives no clear support for using high-dose caffeine protocols in young athletes. Additionally, other moderators, including sex, competitive level, and habitual caffeine intake, were too inconsistently reported or unevenly distributed to support firm conclusions. Their non-significant findings should therefore be interpreted as insufficient evidence of moderation rather than evidence that these characteristics are unimportant [47].

### 4.3. Physical Performance

Physical performance was the most extensively studied domain and provided the clearest finding of this review. In the primary analysis, which excluded a priori the extreme combat-sport count-test effects with implausibly small standard deviations, acute caffeine ingestion produced a small but statistically significant improvement in physical performance across 189 effect sizes from 23 studies (g = 0.33, 95% CI 0.17 to 0.49); including all 193 effects in a sensitivity analysis increased the estimate to g = 0.43, but this value was inflated by the extreme effects. In practical terms, caffeine appeared to help adolescent athletes perform slightly better, but the average benefit was modest rather than dramatic. Favourable effects with confidence intervals excluding the null were observed for change-of-direction and agility performance, aerobic endurance, jumping, sprinting, and acceleration, maximal strength, and muscular endurance. Repeated-sprint ability and anaerobic power also favoured caffeine, although their confidence intervals crossed the null.

Caffeine mainly acts by blocking adenosine receptors, which can increase alertness, reduce central fatigue and support motor-unit recruitment during exercise. It may also influence calcium handling and excitation–contraction coupling within skeletal muscle [1,48,49]. Put more simply, caffeine may help athletes feel more awake and maintain muscular output when exercise becomes uncomfortable or tiring. This explanation is broadly consistent with evidence from adult populations showing small benefits for endurance, sprinting, muscular strength and high-intensity exercise [1,3,50]. However, these mechanisms have mainly been studied in adults and may not have the same effects in adolescents. Differences in maturation, body composition, habitual caffeine intake, and sensitivity to caffeine may influence their responses. Importantly, these favourable effects were confined to general physical-performance measures such as sprinting, jumping, and strength; they should not be read as improvements in sport-specific technical performance or in real competitive outcomes, which are considered separately below.

### 4.4. Sport-Specific Interpretation

Sport-specific performance showed a smaller and less-certain effect than general physical performance. With 50 effect sizes from 11 studies, the pooled estimate favoured caffeine but did not reach statistical significance. Reaction and agility tasks produced the largest sub-outcome estimate, although uncertainty remained considerable, whereas shooting or scoring accuracy showed only a small effect and dribbling performance was essentially unchanged. These results suggest that caffeine may be more helpful for actions depending heavily on movement speed and rapid physical responses than for tasks requiring fine technical control. Because sport, sex, dose, and timing were unevenly distributed across studies, sport-specific conclusions should be treated as hypotheses rather than stable subgroup effects. The adolescent pattern was broadly consistent with adult evidence showing small benefits for endurance and selected high-intensity or strength-related tasks [1,2].

These differences make practical sense. Running faster or maintaining repeated movements mainly depends on physical output and fatigue resistance, whereas passing, shooting, and dribbling also require timing, precision, judgement, and control. Greater alertness may help athletes react more quickly, but excessive arousal, tremor, anxiety, or gastrointestinal discomfort may disrupt skilled performance. Therefore, an improvement in sprinting or jumping should not be assumed to translate directly into better match performance. The available studies were also unevenly distributed across sports, doses, sexes and competitive levels, making it difficult to identify which sporting contexts are most likely to benefit. The non-significant result indicates a lack of clear evidence rather than evidence that caffeine has no effect on sport-specific performance.

### 4.5. Perceptual and Physiological Responses

The perceptual domain grouped subjective responses to the exercise task itselfprincipally rating of perceived exertion, together with task-related pain and fatiguebecause these constructs share a common basis in the conscious perception of effort and discomfort during exercise and are hypothesised to respond to caffeine through the same central pathway, namely reduced adenosine-mediated signalling of effort and pain. Mood and affective states, which reflect a broader psychological construct rather than a task-related perception, were assigned to the separate psychological-response domain. Perceptual responses showed no clear pooled difference between caffeine and placebo. In particular, ratings of perceived exertion did not change reliably. This finding is somewhat different from adult meta-analytic evidence, which generally indicates that caffeine can reduce perceived exertion during exercise [4]. One possible explanation is that the reduction in perceived effort is smaller or less consistent in adolescent athletes. Another is that participants receiving caffeine completed more work while reporting a similar level of exertion. In that situation, an unchanged RPE would not necessarily mean that caffeine had no perceptual effect; athletes may simply have produced more external work for the same perceived effort. Differences in exercise intensity, timing of measurement and the RPE scales used may have further diluted the pooled result [4]. Caffeine can produce noticeable sensations such as alertness, nervousness, increased urination or palpitations. Participants who experience these sensations may correctly guess which condition they received and then report effort in line with their expectations. Conversely, adolescents with little previous caffeine exposure may experience stimulation as discomfort rather than improved readiness. Perceptual findings should therefore be considered together with habitual intake, expectations and the success of blinding, although these factors were poorly reported in the included studies.

Physiological responses were coded according to their biological direction rather than automatically as beneficial or harmful. The pooled estimate was small and non-significant. Heart rate and blood lactate tended to be higher following caffeine under the coding system used, but neither result was statistically reliable. These changes should not be described as performance improvements. A higher heart rate or lactate concentration may reflect acute stimulation, greater exercise output, differences in sampling time or, in some circumstances, a negative physiological response. The physiological domain combined outcomes with different meanings, so its pooled estimate has limited standalone value. Heart rate, blood lactate, blood glucose, blood pressure, and oxygen consumption cannot be placed on a simple “higher is better” or “lower is better” scale, because each reflects a distinct physiological process whose direction of benefit depends on the exercise context. Moreover, several important outcomes, including blood-pressure responses, hydration, sweating, heart-rate variability and recovery markers, were reported too inconsistently to support reliable pooling. The evidence was therefore rated with very low certainty. Overall, caffeine elicited acute physiological responses, but the available evidence does not establish whether these responses are consistently beneficial, harmful, or clinically meaningful in adolescent athletes.

### 4.6. Cognitive and Psychological Outcomes

Cognitive performance showed a small favourable pooled effect, but this result was based on only 15 effect sizes from two study clusters. Stroop interference and simple reaction time both favoured caffeine but did not individually reach statistical significance. The direction is consistent with the known effects of caffeine on alertness, vigilance, and attention, which may be relevant to athletes who must make rapid decisions under fatigue [1,49]. However, the evidence base is simply too small to support a firmer conclusion [1,49,51].

Psychological outcomes were reported by only two study clusters, and most effect sizes were contributed by a single small study. Moreover, the psychological constructs were not consistently replicated across studies, precluding a meaningful domain-level quantitative synthesis. The findings were therefore summarised narratively and provide insufficient evidence to determine whether caffeine improves or worsens mood, motivation, or psychological readiness in adolescent athletes.

### 4.7. Dose and Timing

The available adolescent-athlete data do not support a “higher is better” interpretation. The apparent categorical dose effect was not monotonic and was not supported by a clear continuous dose–response. An exploratory subgroup analysis suggested larger physical-performance estimates when caffeine was ingested 31–60 min before exercise than when it was ingested within 30 min. However, the ≤30 min subgroup was based on only three study clusters, and timing was potentially confounded by dose, sport, administration form, and study design. This finding should therefore be considered hypothesis-generating and should not be interpreted as evidence that 31–60 min is the optimal ingestion window for adolescent athletes. Dosing should be discussed in relative units, because absolute doses such as 400 mg represent different exposures across adolescents of different body mass, and the adult dosing guidance of about 3–6 mg·kg^−1^ should not be transferred directly to minors [52]. If future youth-sport studies test caffeine, lower relative doses, morning administration, careful symptom monitoring, and avoidance of evening use may be more defensible than high-dose protocols; no current result supports 9 mg·kg^−1^ in adolescent athletes [1].

### 4.8. Possible Mechanisms

The adenosine-antagonism pathways outlined in Section 4.3 provide plausible explanations for the small improvements observed in endurance, sprint, jumping, agility, and strength-related tasks. However, the current adolescent-athlete evidence does not directly verify these mechanisms: perceptual response showed no clear pooled difference, and physiological outcomes were not clearly favourable. Mechanistic explanations should therefore remain conditional. This limitation suggests that mechanisms established mainly in adults cannot be assumed to operate with the same magnitude or visibility in adolescents, and it highlights the need for youth-specific mechanistic work.

### 4.9. Strengths and Limitations

This review has several strengths. It focused specifically on adolescent athletes, used three-level models to account for dependent effect sizes, applied cluster-robust inference, and considered both performance and safety outcomes. These methods help reduce overconfidence that can arise when multiple outcomes from the same trial are treated as independent. Several limitations remain. The number of included studies was modest, and many had small samples. Most used crossover designs and contributed multiple dependent effect sizes, requiring modelling assumptions about within-participant correlation and effect-size dependence. Although sensitivity analyses supported the direction of the physical-performance effect, this domain showed high heterogeneity, wide prediction intervals, and small-study effects, and several outcomes, including cognitive and psychological responses, were informed by only two study clusters. The review also searched only PubMed and Web of Science, used English-language restrictions, and did not include trial registries or the grey literature. Several included trials used multi-ingredient products (energy or sports drinks), in which the caffeine effect cannot be fully isolated from co-ingredients despite our matching requirement. The moderator tests in sparse domains should be regarded as exploratory. In particular, the ingestion-timing contrast was highly unbalanced, with only three independent clusters in the ≤30 min category, precluding a reliable conclusion about optimal timing. The included reports varied in sport, test type, dose, timing, administration form, and competitive level. As quantified in Section 3.2, biological or pubertal maturity was not reported in 28 of 32 reports, habitual caffeine intake was not reported in 7 of 32 reports, and menstrual status was not reported in 3 of the 9 reports that included female participants. Training age and sleep were also inconsistently reported. These reporting limitations contributed to concerns regarding the indirectness of the evidence [53]. Accordingly, this review cannot evaluate chronic caffeine use, repeated use during competition, long-term safety, the normalisation of supplement use, or developmental consequences.

### 4.10. Safety, Ethical Boundaries, and Practical Implications

The available acute evidence did not reveal a serious safety signal, but it is not sufficient to establish safety in minors. Most included studies were small, follow-up was short, adverse-event instruments were inconsistent, and several studies did not systematically assess symptoms. Sleep effects may occur after the performance test and may not be captured by immediate post-exercise monitoring, which matters because sleep is central to growth, recovery, learning, and injury-risk management in adolescent athletes. The safety signals that were reported point to plausible risk modifiers: higher doses, especially 9 mg·kg^−1^, were associated with more gastrointestinal symptoms, headache, insomnia, and palpitations, and evening ingestion appeared to increase insomnia, headache, tachycardia, and diuresis. Energy drinks and caffeinated sports drinks complicate interpretation because they may contain sugar, taurine, guarana, ginseng, or other bioactive ingredients, and their effects should not be attributed to caffeine alone unless the comparator is matched for non-caffeine ingredients. Ethically, caffeine use in minors is not a purely individual performance choice; it involves athlete assent, parental or guardian consent, coach influence, sport-organisation policy, medical oversight, and the risk of normalising supplement use early in athletic development. The IOC supplement consensus recommends that performance supplements be considered only when evidence supports safety, legality, and effectiveness, and ideally after expert assessment of needs and risks standards that are harder to satisfy in minors, in whom safety evidence is weaker and the athlete may be more vulnerable to external pressure. In this setting, incomplete evidence should lead to tighter safeguards, not to a claim that caffeine is either proven safe or categorically unsafe. These findings apply only to acute, experimentally controlled exposures in adolescent athletes and should not be extrapolated to children, non-athlete adolescents, repeated or chronic use, or energy drinks.

Current evidence suggests that acute caffeine may produce small favourable effects on selected physical-performance tasks in adolescent athletes, but certainty is low, heterogeneity is high, small-study effects are present, and safety monitoring is incomplete; the evidence therefore does not support broad or routine recommendation of caffeine for adolescent athletes [44,54]. If caffeine use is considered in a specific high-performance youth setting, it should be treated as a monitored clinical and sport-nutrition decision rather than a general training strategy, with assessment of age, body mass, maturity, sleep pattern, anxiety symptoms, cardiac history, medication use, habitual caffeine intake, competition timing, parental consent, athlete assent, and previous adverse responses [45,55]. High-dose caffeine, evening ingestion, and energy drinks should be avoided. For most youth-sport settings, the default position should remain food-first, sleep protection, hydration, adequate carbohydrate availability, and training quality [56].

### 4.11. Future Directions

Future trials should be designed specifically for adolescent athletes rather than adapted from adult protocols. Studies should include larger samples and stratify or adjust for age, sex, pubertal stage, biological maturity, sport type, competitive level, and habitual caffeine intake. Caffeine dose should be standardised in mg·kg^−1^, administration timing should be pre-specified, and capsules, coffee, chewing gum, mouth rinse, and energy drinks should be distinguished, with multi-ingredient products not interpreted as caffeine effects unless non-caffeine ingredients are matched in the comparator. Studies should systematically record adverse events, sleep latency and duration, anxiety, palpitations, blood pressure, heart rate, gastrointestinal symptoms, headache, tremor, dizziness, and next-day recovery, and studies of female athletes should report menstrual status, iron status, and indicators of energy availability. Crossover trials should report washout, sequence and period effects, paired means, change scores, and within-participant correlations. Future work should develop a youth-athlete risk–benefit framework that prioritises health, consent, and sleep before short-term performance change.

## 5. Conclusions

Acute caffeine ingestion may produce small, favourable effects on selected physical-performance outcomes in adolescent athletes. Evidence for sport-specific skills, perceptual responses, physiological responses, psychological responses, and cognitive performance remains uncertain or very uncertain, and the certainty of the physical-performance estimate itself is low. The available trials do not establish the safety of acute caffeine exposure across adolescent athletes: no serious adverse events were reported, but adverse-event monitoring was inconsistent, sleep outcomes were incompletely assessed, and follow-up was short. These findings do not support routine caffeine recommendation in youth sport and should not be extrapolated to children, non-athlete adolescents, chronic use, or energy-drink consumption.

## Figures and Tables

**Table 1:** GRADE summary of findings by outcome domain.

Outcome Domain	Studies/Effects	Effect: g (95% CI); PI	GRADE	Interpretation
Physical performance	23/189	0.33 (0.17 to 0.49), primary analysis (extreme combat-sport effects excluded a priori); all effects: 0.43 (0.20 to 0.67), PI −0.71 to 1.58	Low	Caffeine may produce a small improvement in overall physical performance.
Sport-specific performance	11/50	0.23 (−0.05 to 0.50); PI −0.67 to 1.13	Very low	Caffeine may have little or no effect on sport-specific performance; evidence very uncertain.
Physiology	10/34	−0.14 (−0.35 to 0.07); PI −0.92 to 0.64	Very low	Caffeine may slightly raise HR/lactate (non-significant); evidence very uncertain.
Perceptual response	13/45	0.16 (−0.16 to 0.48); PI −1.17 to 1.50	Very low	Caffeine may have little or no effect on perceived exertion; evidence very uncertain.
Psychological response	2/26	0.91 (−2.60 to 4.43); PI −5.67 to 7.50	Very low	Not a stable pooled estimate; shown for transparency only and interpreted narratively (see Note ^^a^).
Cognitive performance	2/15	0.32 (0.06 to 0.58); PI −2.67 to 3.30	Very low	Caffeine may improve cognition, but evidence very uncertain (2 studies).

Note. All estimates start at high certainty and are downgraded by GRADE domains. Physical performance (low certainty): −1 for risk of bias (RoB 2 predominantly “some concerns”), −1 for inconsistency (*I*^2^ = 84% with a wide prediction interval spanning zero), and −1 for publication bias (significant cluster-robust funnel asymmetry indicating small-study effects); not downgraded for indirectness because participants were adolescent athletes. Sport-specific performance, perceptual response, and physiology (very low): −1 for risk of bias, −1 for inconsistency, and −2 for imprecision (confidence intervals crossing the null with few clusters). Psychological response and cognitive performance (very low): downgraded for very serious imprecision because each domain was informed by only two study clusters with very wide prediction intervals. ^^a^ The pooled estimate for psychological response is presented for transparency only and must not be interpreted as a reliable domain-level effect: it rests on only two study clusters, with 20 of the 26 effect sizes contributed by a single study and substantial between-effect heterogeneity (*I*^2^ = 94%), so the confidence interval (−2.60 to 4.43) and prediction interval (−5.67 to 7.50) are uninformative. This domain was therefore synthesised narratively (Section 3.5.3), and no inference about the direction or magnitude of a caffeine effect on psychological outcomes should be drawn from this row. A pooled estimate is reported for cognitive performance because its effects were more evenly distributed across the two clusters, whereas the psychological effects were dominated by a single cluster. CI, confidence interval; PI, prediction interval; RoB 2, Cochrane risk-of-bias tool version 2.

## Data Availability

The effect-size extraction dataset, data dictionary, and complete analysis code (R; metafor and clubSandwich) supporting the conclusions of this article are openly available on the Open Science Framework (https://osf.io/t2ea3 (accessed on 1 June 2026); https://doi.org/10.17605/OSF.IO/PXAGY), where the study protocol was also prospectively registered. These materials reproduce all reported effect sizes, the three-level meta-analytic models, sensitivity analyses, and figures.

## Supplementary Materials

The following supporting information can be downloaded at: https://www.mdpi.com/article/10.3390/nu18152520/s1, Table S1: PRISMA 2020 checklist; Table S2: Search strategy; Table S3: Risk-of-bias assessment using RoB 2; Table S4: PEDro scale scores; Table S5: Study characteristics; Table S5A: Age characteristics and eligibility assessment of included studies; Table S6: Safety and adverse-event findings of the included studies; Table S7: Small-study effect (Egger-type) regression diagnostics by outcome domain; Supplementary Note S1. Effect-size calculation.
