# Supplementary material for: Acute Effects and Safety of Caffeine Ingestion in Adolescent Athletes: A Systematic Review and Three-Level Meta-Analysis
Source: Nutrients. 2026 Aug 3;18(15):2520. doi: 10.3390/nu18152520 (PMC13468084; doi:10.3390/nu18152520)
Supplement: Supplementary file 1 [file nutrients-18-02520-s001.zip › nutrients-4423320-supplementary.pdf]

nutrients

MDPI

Supplementary materials

Supplementary Table 1. PRISMA 2020 checklist.

Section and Topic	Item #	Checklist item	Location where item is reported
**TITLE Acute Effects and Safety of Caffeine Ingestion in Adolescent Athletes: A Systematic Review and Three-Level Meta-Analysis**			Page 1
Title	1	Identify the report as a systematic review.	Page 1
**ABSTRACT**			
Abstract	2	See the PRISMA 2020 for Abstracts checklist.	Page 1
**INTRODUCTION**			
Rationale	3	Describe the rationale for the review in the context of existing knowledge.	Page 2
Objectives	4	Provide an explicit statement of the objective(s) or question(s) the review addresses.	Page 3
**METHODS**			
Eligibility criteria	5	Specify the inclusion and exclusion criteria for the review and how studies were grouped for the syntheses.	Pages 3–4
Information sources	6	Specify all databases, registers, websites, organisations, reference lists and other sources searched or consulted to identify studies. Specify the date when each source was last searched or consulted.	Page 4
Search strategy	7	Present the full search strategies for all databases, registers and websites, including any filters and limits used.	Page 4
Selection process	8	Specify the methods used to decide whether a study met the inclusion criteria of the review, including how many reviewers screened each record and each report retrieved, whether they worked independently, and if applicable, details of automation tools used in the process.	Pages 4–5
Data collection process	9	Specify the methods used to collect data from reports, including how many reviewers collected data from each report, whether they worked independently, any processes for obtaining or confirming data from study investigators, and if applicable, details of automation tools used in the process.	Page 5

Data items	10a	List and define all outcomes for which data were sought. Specify whether all results that were compatible with each outcome domain in each study were sought (e.g. for all measures, time points, analyses), and if not, the methods used to decide which results to collect.	Pages 5–6
	10b	List and define all other variables for which data were sought (e.g. participant and intervention characteristics, funding sources). Describe any assumptions made about any missing or unclear information.	Page 4
Study risk of bias assessment	11	Specify the methods used to assess risk of bias in the included studies, including details of the tool(s) used, how many reviewers assessed each study and whether they worked independently, and if applicable, details of automation tools used in the process.	Page 5
Effect measures	12	Specify for each outcome the effect measure(s) (e.g. risk ratio, mean difference) used in the synthesis or presentation of results.	Page 5
Synthesis methods	13a	Describe the processes used to decide which studies were eligible for each synthesis (e.g. tabulating the study intervention characteristics and comparing against the planned groups for each synthesis (item #5)).	Pages 5–6
	13b	Describe any methods required to prepare the data for presentation or synthesis, such as handling of missing summary statistics, or data conversions.	Page 5
	13c	Describe any methods used to tabulate or visually display results of individual studies and syntheses.	Page 6
	13d	Describe any methods used to synthesize results and provide a rationale for the choice(s). If meta-analysis was performed, describe the model(s), method(s) to identify the presence and extent of statistical heterogeneity, and software package(s) used.	Pages 6–20
	13e	Describe any methods used to explore possible causes of heterogeneity among study results (e.g. subgroup analysis, meta-regression).	Pages 5–6
	13f	Describe any sensitivity analyses conducted to assess robustness of the synthesized results.	Pages 5–6
Reporting bias assessment	14	Describe any methods used to assess risk of bias due to missing results in a synthesis (arising from reporting biases).	Page 6
Certainty assessment	15	Describe any methods used to assess certainty (or confidence) in the body of evidence for an outcome.	Page 6

**RESULTS**			
Study selection	16a	Describe the results of the search and selection process, from the number of records identified in the search to the number of studies included in the review, ideally using a flow diagram.	Page 7
	16b	Cite studies that might appear to meet the inclusion criteria, but which were excluded, and explain why they were excluded.	Page 7
Study characteristics	17	Cite each included study and present its characteristics.	Page 7;

			Supplementary Table 5
Risk of bias in studies	18	Present assessments of risk of bias for each included study.	Page 8; Supplementary Table 3
Results of individual studies	19	For all outcomes, present, for each study: (a) summary statistics for each group (where appropriate) and (b) an effect estimate and its precision (e.g. confidence/credible interval), ideally using structured tables or plots.	Pages 8–20
Results of syntheses	20a	For each synthesis, briefly summarise the characteristics and risk of bias among contributing studies.	Pages 8–20
	20b	Present results of all statistical syntheses conducted. If meta-analysis was done, present for each the summary estimate and its precision (e.g. confidence/credible interval) and measures of statistical heterogeneity. If comparing groups, describe the direction of the effect.	Pages 8–20
	20c	Present results of all investigations of possible causes of heterogeneity among study results.	Pages 9–20
	20d	Present results of all sensitivity analyses conducted to assess the robustness of the synthesized results.	Pages 12–13
Reporting biases	21	Present assessments of risk of bias due to missing results (arising from reporting biases) for each synthesis assessed.	Pages 12–13
Certainty of evidence	22	Present assessments of certainty (or confidence) in the body of evidence for each outcome assessed.	Pages 13–14
**DISCUSSION**			
Discussion	23a	Provide a general interpretation of the results in the context of other evidence.	Pages 21–24
	23b	Discuss any limitations of the evidence included in the review.	Page 23
	23c	Discuss any limitations of the review processes used.	Page 23
	23d	Discuss implications of the results for practice, policy, and future research.	Pages 23–24
**OTHER INFORMATION**			
Registration and protocol	24a	Provide registration information for the review, including register name and registration number, or state that the review was not registered.	Page 3
	24b	Indicate where the review protocol can be accessed, or state that a protocol was not prepared.	Page 3
	24c	Describe and explain any amendments to information provided at registration or in the protocol.	Page 3
Support	25	Describe sources of financial or non-financial support for the review, and the role of the funders or sponsors in the review.	Not reported
Competing interests	26	Declare any competing interests of review authors.	Not reported
Availability of data,	27	Report which of the following are publicly available and where they can be found: template data collection forms; data extracted	Not reported

code and other materials		from included studies; data used for all analyses; analytic code; any other materials used in the review.	

Supplementary Table 2. Search strategy

**Pubmed(170) Search date:13 May 2026**<br>adolescent[MeSH Terms]OR adolescen*OR youth*OR teen*OR teenager*OR pediatricOR paediatricOR juvenileOR "young athlete*"OR "under-18"OR "under 18"OR U18OR "U-18"OR U17OR "U-17"OR U16OR "U-16"OR U15OR "U-15")AND(athlete*OR player*OR "young athlete*"OR "youth sport*"OR academyOR soccerOR footballOR basketballOR volleyballOR handballOR rugbyOR hockeyOR futsalOR swimmingOR cyclingOR runningOR combatOR tennis)AND(caffeine[MeSH Terms]OR caffeineOR caffeinatedOR coffeeOR espressoOR "energy drink*"OR "caffeine gum"OR "caffeinated gum"OR "caffeine gel"OR "caffeine mouth rinse"OR "1,3,7-trimethylxanthine")AND(performanceOR ergogenicOR sprintOR jumpOR agilityOR enduranceOR strengthOR powerOR skillOR shootingOR passingOR cognitionOR decision-makingOR reaction timeOR fatigueOR safetyOR "adverse event*"OR insomniaOR sleepOR tachycardiaOR anxietyOR headache)AND(randomized controlled trial[Publication Type]OR random*OR placeboOR crossoverOR "cross-over"OR "double-blind"OR "single-blind"OR trial)NOT(animal[mh]NOT human[mh])
**Web of science(359) Search date:13 May 2026**<br>TS=((adolescen*OR youth*OR teen*OR teenager*OR pediatricOR paediatricOR juvenileOR juniorOR academyOR "young athlete*"OR schoolboy*OR schoolgirl*OR "under-18"OR "under 18"OR U18OR U17OR U16OR U15)AND(athlete*OR player*OR sport*OR soccerOR footballOR basketballOR volleyballOR handballOR rugbyOR hockeyOR futsalOR swimmingOR cyclingOR runningOR tennisOR badmintonOR taekwondoOR judoOR combatOR gymnast*OR athletics)AND(caffeineOR caffeinatedOR coffeeOR espressoOR "energy drink*"OR "caffeine gum"OR "caffeine gel"OR "caffeine mouth rinse"OR "1,3,7-trimethylxanthine"))
**SPORTDiscus(75) Search date:15 July 2026**<br>S1：TI (adolescen* OR youth* OR teen* OR teenager* OR pediatric OR paediatric OR juvenile OR (young N1 athlete*) OR (young N1 player*) OR "under 18" OR "under-18" OR U18 OR "U-18" OR U17 OR "U-17" OR U16 OR "U-16" OR U15 OR "U-15") OR AB (adolescen* OR youth* OR teen* OR teenager* OR pediatric OR paediatric OR juvenile OR (young N1 athlete*) OR (young N1 player*) OR "under 18" OR "under-18" OR U18 OR "U-18" OR U17 OR "U-17" OR U16 OR "U-16" OR U15 OR "U-15")<br>S2：TI (athlete* OR player* OR sport* OR soccer OR football OR basketball OR volleyball OR handball OR rugby OR hockey OR futsal OR swim* OR cycl* OR run* OR runner* OR tennis OR badminton OR taekwondo OR judo OR gymnast* OR sprint* OR "track and field") OR AB (athlete* OR player* OR sport* OR soccer OR football OR basketball OR volleyball OR handball OR rugby OR hockey OR futsal OR swim* OR cycl* OR run* OR runner* OR tennis OR badminton OR taekwondo OR judo OR gymnast* OR sprint* OR "track and field")<br>S3：TI (caffeine OR caffeinated OR coffee OR espresso OR (energy N1 drink*)OR "caffeine gum*" OR "caffeinated gum*" OR "caffeine gel*"OR "caffeine mouth rinse*" OR "1,3,7-trimethylxanthine")OR AB (caffeine OR caffeinated OR coffee OR espresso OR (energy N1 drink*)OR "caffeine gum*" OR "caffeinated gum*" OR "caffeine gel*"OR "caffeine mouth rinse*" OR "1,3,7-trimethylxanthine")<br>S4 = S1 AND S2 AND S3

Supplementary Table 3. RoB 2 risk-of-bias assessment.

Study	D1	D2	D3	D4	D5	Overall
Sasaki et al., 1987	Some concerns	Some concerns	Low	Low	Some concerns	Some concerns
Collomp et al., 1992	Some concerns	Some concerns	Low	Low	Some concerns	Some concerns
Guttierres et al., 2013	Some concerns	Some concerns	Low	Some concerns	Some concerns	Some concerns
Abian-Vicen et al., 2014	Some concerns	Low	Low	Low	Some concerns	Some concerns
Jordan et al., 2014	Low	Low	Low	Low	Some concerns	Some concerns
Pettersen et al., 2014	Some concerns	Some concerns	Some concerns	Low	Some concerns	Some concerns
Gallo-Salazar et al., 2015	Some concerns	Low	Low	Low	Some concerns	Some concerns
Arazi et al., 2016	Some concerns	Some concerns	Low	Some concerns	Some concerns	Some concerns
Astley et al., 2017	Some concerns	Low	Low	Low	Some concerns	Some concerns
Ellis et al., 2019	Some concerns	Some concerns	Low	Low	Some concerns	Some concerns
Scanlan et al., 2019	Some concerns	Low	Low	Low	Some concerns	Some concerns
Russell et al., 2020	Some concerns	Low	Low	Low	Some concerns	Some concerns
Egesoy et al., 2020	Some concerns	High	Low	High	Some concerns	High

Spineli et al., 2020	Some concerns	Low	Some concerns	Low	Some concerns	Some concerns
Stojanović et al., 2022	Some concerns	Low	Low	Low	Some concerns	Some concerns
Delleli et al., 2023	Some concerns	Some concerns	Low	Some concerns	Some concerns	Some concerns
Dos Santos et al., 2023	Some concerns	Low	Low	Low	Some concerns	Some concerns
Bougrine et al., 2023	Some concerns	Some concerns	Some concerns	Low	Some concerns	Some concerns
Ouergui et al., 2023	Some concerns	Some concerns	Low	Some concerns	Some concerns	Some concerns
Spineli et al., 2024	Some concerns	Some concerns	Low	Some concerns	Some concerns	Some concerns
Ghazaleh et al., 2024	Some concerns	Low	Low	Some concerns	Some concerns	Some concerns
Bougrine et al., 2024a	Low	Low	Low	Some concerns	Some concerns	Some concerns
Bougrine et al., 2024b	Some concerns	Some concerns	Low	Low	Some concerns	Some concerns
Sánchez-Redondo et al., 2025	Low	Low	Low	Low	Some concerns	Some concerns
Bezuglov et al., 2025a	Some concerns	Some concerns	Low	Some concerns	Some concerns	Some concerns
Bezuglov et al., 2025b	Some concerns	Some concerns	Low	Some concerns	Some concerns	Some concerns
Bougrine et al., 2025	Some concerns	Some concerns	Low	Some concerns	Some concerns	Some concerns

Miraftabi et al., 2025	Some concerns	Some concerns	Low	Some concerns	Some concerns	Some concerns
Hemmat et al., 2025	Some concerns	Low	Low	Low	Some concerns	Some concerns
Amor et al., 2025	Some concerns	Some concerns	Low	Some concerns	Some concerns	Some concerns
Alejo et al., 2026	Some concerns	Low	Low	Low	Some concerns	Some concerns
Yasli et al., 2026	Some concerns	Some concerns	Low	Some concerns	Some concerns	Some concerns

Supplementary Table 4. PEDro methodological-quality assessment.

Study	The first author	Year of Publication	D1	D2	D3	D4	D5	D6	D7	D8	D9	D10	D11	Total
1	Sasaki et al., 1987	1987	Y	0	0	0	1	1	0	1	1	1	1	6
2	Collomp et al., 1992	1992	Y	1	0	0	1	1	0	1	1	1	1	7
3	Guttierres et al., 2013	2013	Y	0	0	0	1	1	0	1	1	1	1	6
4	Abian-Vicen et al., 2014	2014	Y	1	0	0	1	1	1	1	1	1	1	8
5	Jordan et al., 2014	2014	Y	1	0	0	1	1	0	1	1	1	1	7
6	Pettersen et al., 2014	2014	Y	1	0	0	1	1	0	1	1	1	1	7
7	Gallo-Salazar et al., 2015	2015	Y	1	0	0	1	1	1	1	1	1	1	8
8	Arazi et al., 2016	2016	Y	1	0	0	1	1	0	1	1	1	1	7
9	Astley et al., 2017	2017	Y	1	0	0	1	1	0	1	1	1	1	7

10	Ellis et al., 2019	2019	Y	1	0	0	1	0	0	1	1	1	1	6
11	Scanlan et al., 2019	2019	Y	1	0	0	1	0	1	1	1	1	1	7
12	Russell et al., 2020	2020	Y	1	0	0	1	0	1	1	1	1	1	7
13	Egesoy et al., 2020	2020	Y	1	0	0	1	0	0	1	1	1	1	6
14	Spineli et al., 2020	2020	Y	1	0	0	1	1	1	1	0	1	1	7
15	Stojanović et al., 2022	2022	Y	1	0	0	1	1	1	1	1	1	1	8
16	Delleli et al., 2023	2023	Y	1	0	0	1	0	1	1	1	1	1	7
17	Dos Santos et al., 2023	2023	Y	1	0	0	1	1	0	1	1	1	1	7
18	Bougrine et al., 2023	2023	Y	1	0	0	1	1	0	0	0	1	1	5
19	Ouergui et al., 2023	2023	Y	1	0	0	1	1	0	1	1	1	1	7
20	Spineli et al., 2024	2024	Y	1	0	0	1	1	0	1	1	1	1	7
21	Ghazaleh et al., 2024	2024	Y	1	0	0	1	1	0	1	1	1	1	7
22	Bougrine et al., 2024a	2024a	Y	1	0	0	1	1	0	1	1	1	1	7
23	Bougrine et al., 2024b	2024b	Y	1	0	0	1	1	1	1	1	1	1	8
24	Sánchez-Redondo et al., 2025	2025	Y	1	0	0	1	1	1	1	1	1	1	8
25	Bezuglov et al., 2025a	2025a	Y	1	0	1	1	1	0	1	0	1	1	7
26	Bezuglov et al., 2025b	2025b	Y	1	0	1	1	1	0	1	0	1	1	7

27	Bougrine et al., 2025	2025	Y	1	0	0	1	1	0	1	1	1	1	7
28	Miraftabi et al., 2025	2025	Y	1	0	0	1	1	0	1	1	1	1	7
29	Hemmat et al., 2025	2025	Y	1	0	0	1	1	1	1	1	1	1	8
30	Amor et al., 2025	2025	Y	1	1	0	1	1	1	1	1	1	1	9
31	Alejo et al., 2026	2026	Y	1	0	0	1	1	1	1	1	1	1	8
32	Yasli et al., 2026	2026	Y	1	0	0	1	1	0	1	1	1	1	7

Supplementary Table 5. Characteristics of included studies.

Study (year)	Sport	Age	Sex	Level	Design	Dose	Timing	n	Outcomes contributed (effects)
Sasaki et al., 1987	running	15.6±0.4	Male	Trained	crossover	6.64 mg/kg	60 min pre	5	Physical performance (2)
Collomp et al., 1992	swimming	17±2.1	Mixed	Trained	crossover	4.3 mg/kg	2 h after meal	7	Physiology (4)
Guttierres et al., 2013	Soccer	16.1±0.7	Male	Trained	crossover	7.2 mg/kg	20 min pre	20	Perceptual response (2); Physiology (1)
Abian-Vicen et al., 2014	Basketball	14.9±0.8	Male	Elite	crossover	3mg/kg	60 minutes	16	Physical performance (4); Sport-specific performance (2)
Jordan et al., 2014	Soccer	14.1±0.5	Male	Elite	crossover	6mg/kg	1 h pre	17	Sport-specific performance (6); Perceptual response (2); Physiology (1)
Pettersen et al., 2014	Soccer	17.6±1.1	Male	Elite	crossover	6mg/kg	50 min before warm-up	21	Physiology (10); Sport-specific performance (7); Perceptual response (1)
Gallo-Salazar et al., 2015	Tennis	16±1	Mixed	Elite	crossover	3mg/kg	60 min pre	14	Sport-specific performance (13); Physical performance (5); Physiology (2)
Arazi et al., 2016	Karate	16.8±1.23	Female	Trained	crossover	2 mg/kg	60 min pre	10	Perceptual response (16); Physical performance (8)
Astley et al., 2017	Judo	16.1±1.4	Male	Elite	crossover	4 mg/kg	60 min pre	18	Physiology (3); Perceptual response (1); Sport-specific performance (1); Physical performance (1)

Ellis et al., 2019	Soccer	16±1	Male	Elite	crossove r	1 mg/kg	60 min pre	15	Physical performance (27)
Scanlan et al., 2019	Basketball	18.3±3.3	Mixed	Elite	crossove r	3 mg/kg	60 min pre	21	Sport-specific performance (6)
Russell et al., 2020	Rugby union	18±1	Male	Elite	crossove r	4.1 mg/kg	During simulated half-time; ~10 min before RSSA2	14	Physical performance (6); Cognitive performance (3)
Egesoy et al., 2020	Soccer	16.5±0.5	Male	Elite	crossove r	6 mg/kg	60 min pre-exercise	48	Sport-specific performance (3); Physical performance (1)
Spineli et al., 2020	Volleyball / soccer / track and field	15±2	NR	Trained	crossove r	6 mg/kg	60 min pre-exercise	10 0	Physical performance (7); Perceptual response (1)
Stojanović et al., 2022	Basketball	16.5±1	Male	Elite	crossove r	3 mg/kg	60 min pre-exercise; morning 10:00	11	Physical performance (16); Sport-specific performance (2)
Delleli et al., 2023	taekwondo	18.25±0.75	Male	Elite	crossove r	3 mg/kg	60 min pre exercise; warm-up 50 min after supplementation	16	Psychological response (20); Physical performance (6); Perceptual response (6)
Dos Santos et al., 2023	athletics/volleyball/soc cer	15.5±2	Male	Trained	crossove r	6 mg/kg	60 min pre exercise	90	Physical performance (7)
Bougrine et al., 2023	Handball	16.6±0.5	Femal e	Elite	crossove r	6 mg/kg	60 min pre-exercise	20	Physical performance (8)
Ouergui et al., 2023	taekwondo	Elite(M:18.2±0.8, F:17.7±0.6);Sub (M:17.7±0.8, F:17.3±0.5)	Mixed	Elite	crossove r	3 mg/kg	45 min pre warm-up; tests after standardized warm-up	16	Physical performance (11); Perceptual response (4)
Spineli et al., 2024	mixed: volleyball/track and field/soccer	DD(16±1.7); DI(16±2); II(15±1.7)	Male	Trained	crossove r	6 mg/kg	60 min pre	40	Physiology (6); Physical performance (3); Perceptual response (3)
Ghazaleh et al., 2024	futsal	15.9±1.2	Male	Elite	crossove r	6 mg/kg	60 min pre	10	Physical performance (3)
Bougrine et al., 2024a	mixed team sports: handball/football	16.9±0.6	Femal e	Trained	crossove r	3 mg/kg	60 min pre	16	Physical performance (12); Perceptual response (3)
Bougrine et al., 2024b	handball	18.3±0.5	Femal e	Trained	crossove r	3 mg/kg	60 min pre; 6:00 p.m.	15	Physical performance (8); Perceptual response (2)
Sánchez-Redondo et	cycling	17±1	Male	Elite	crossove	3 mg/kg	during	11	Physiology (2); Physical

al., 2025					r		prolonged cycling; ~60 min before TT		performance (1)
Bezuglov et al., 2025a†	soccer	16.5±0.89	Male	Elite	parallel	5.67 mg/kg median (G1); 5.65 mg/kg median (G2)	60 min pre	54	Physical performance (36); Sport-specific performance (1)
Bezuglov et al., 2025b†	soccer	16.5±0.89	Male	Elite	parallel	5.67 mg/kg median ; 5.65 mg/kg median (G2)	60 min pre	54	Perceptual response (3); Physiology (3)
Bougrine et al., 2025	handball	16.76±0.4	Female	Trained	crossover	6 mg/kg	50 min before warm-up; tests after ~10 min warm-up	17	Physical performance (4); Perceptual response (1)
Miraftabi et al., 2025	soccer	18.1±0.9	Male	Trained	crossover	NR	fasted; eight 15-s mouth rinses before tests	13	Physical performance (10)
Hemmat et al., 2025	basketball	16.7±0.86	Male	Recreational	crossover	3 mg/kg	15 min pre performance test	20	Physical performance (6); Sport-specific performance (4)
Amor et al., 2025	volleyball	16.9±0.7	Male	Trained	crossover	3 mg/kg	30 min pre cognitive test	24	Cognitive performance (12)
Alejo et al., 2026	cycling	16.3±0.5	Male	Trained	crossover	6 mg/kg	60 min before 8-min TT	13	Psychological response (6); Physiology (2); Physical performance (1)
Yasli et al., 2026	soccer	14.07±0.26	Male	Elite	crossover	3 mg/kg	45 min pre	14	Sport-specific performance (5)

Note:Sub-Sub-elite; † Companion reports from the same cohort; participants were counted once in the total sample size.

Supplementary Table 5. Characteristics of included studies.(continued)

Study (year)	Habitual caffeine intake	Pubertal / biological maturity	Menstrual status
Sasaki et al., 1987	NR	NR	NR
Collomp et al., 1992	caffeine prohibited 1 week	NR	NR
Guttierres et al., 2013	13 low (<50 mg/day); 5 moderate (50–200 mg/day); 2 regular (~200 mg/day)	NR	NR
Abian-Vicen et al., 2014	NR	NR	NR
Jordan et al., 2014	53.2 ± 64.6 mg/day	Tanner stage V	NR
Pettersen et al., 2014	NR	NR	NR
Gallo-Salazar et al., 2015	light caffeine consumers	NR	NR
Arazi et al., 2016	light caffeine consumers	NR	NR
Astley et al., 2017	NR	NR	NR
Ellis et al., 2019	mean habitual caffeine intake 43 mg/day	NR	NR
Scanlan et al., 2019	light caffeine consumers (<100 mg/day)	NR	Luteal phase
Russell et al., 2020	191 ± 138 mg/day	NR	NR

Egesoy et al., 2020	NR	NR	NR
Spineli et al., 2020	48.8 ± 41.7 mg/day	NR	NR
Stojanović et al., 2022	Light consumers: 310 ± 76 mg/week	NR	NR
Delleli et al., 2023	low caffeine consumers; 1.33 ± 0.42 mg/kg/day	NR	NR
Dos Santos et al., 2023	50.9 ± 40.3 mg/day	NR	NR
Bougrine et al., 2023	56.2 ± 21.1 mg/day	NR	Follicular and/or luteal phase
Ouergui et al., 2023	mild caffeine consumers; 1.14 ± 0.49 mg/kg/day	NR	Early follicular phase
Spineli et al., 2024	NR	NR	NR
Ghazaleh et al., 2024	daily caffeine consumption <70 mg/day	Post-pubertal	NR
Bougrine et al., 2024a	Mild CAF consumers; 1.09 ± 0.3 mg/kg/day	NR	Regular cycles; follicular and/or luteal phase
Bougrine et al., 2024b	Low CAF consumers; 0.78 ± 0.2 mg/kg/day; >25 mg/day	NR	Regular cycles; follicular and/or luteal phase

Sánchez-Redondo et al., 2025	39.5 ± 61.1 mg/day; 0.6 ± 1.0 mg/kg/day	NR	NR
Bezuglov et al., 2025a	low habitual caffeine intake	98.6% predicted adult height	NR
Bezuglov et al., 2025b	low habitual caffeine intake	98.6% predicted adult height	NR
Bougrine et al., 2025	43.55 ± 10.67 mg/day; 0.73 ± 0.15 mg/kg/day	NR	Regular cycles; follicular and/or luteal phase
Miraftabi et al., 2025	NR	NR	NR
Hemmat et al., 2025	excluded if >300 mg/day	NR	NR
Amor et al., 2025	1.14 ± 0.11 mg/kg/day; mild caffeine users	NR	NR
Alejo et al., 2026	naive consumers (<25 mg/day)	NR	NR
Yasli et al., 2026	naive consumers (<25 mg/day)	NR	NR

Supplementary Table 5A. Age characteristics and eligibility assessment of included studies.

Study (year)	Reported age	Reported age range/eligibility range	Statistic
Sasaki et al., 1987	15.6 ± 0.4	NR	Mean ± SD
Collomp et al., 1992	17	NR	Mean; SEM 2.1 (overall n = 14)
Guttierres et al., 2013	16.1 ± 0.7	15–17	Mean ± SD
Abian-Vicen et al., 2014	14.9 ± 0.8	NR	Mean ± SD
Jordan et al., 2014	14.1 ± 0.5	NR	Mean ± SD
Pettersen et al., 2014	17.6 ± 1.1	NR	Mean ± SD (full sample n = 22)
Gallo-Salazar et al., 2015	16.36 ± 1.15	NR	Mean ± SD
Arazi et al., 2016	16.8 ± 1.23	NR	Mean ± SD
Astley et al., 2017	16.1 ± 1.4	14–17	Mean ± SD
Ellis et al., 2019	16 ± 1	NR	Mean ± SD
Scanlan et al., 2019	18.3 ± 3.3	NR	Mean ± SD
Russell et al., 2020	18 ± 1	NR	Mean ± SD

Egesoy et al., 2020	16.5 ± 0.5	NR	Mean ± SD
Spineli et al., 2020	15 ± 2	NR	Mean ± SD
Stojanović et al., 2022	16.5 ± 1	All participants <18	Mean ± SD
Delleli et al., 2023	18.25 ± 0.75	Eligibility: ≥17	Mean ± SD
Dos Santos et al., 2023	15.5 ± 2	12–18 (eligibility)	Mean ± SD
Bougrine et al., 2023	16.6 ± 0.5	16–17	Mean ± SD
Ouergui et al., 2023	Elite: M 18.2 ± 0.8; F 17.7 ± 0.6; sub-elite: M 17.7 ± 0.8; F 17.3 ± 0.5	NR	Subgroup means ± SD
Spineli et al., 2024	DD 16 ± 1.7; DI 16 ± 2.0; II 15 ± 1.7	NR	Genotype-group means ± SD
Ghazaleh et al., 2024	15.9 ± 1.2	NR	Mean ± SD
Bougrine et al., 2024a	16.9 ± 0.6	16–18	Mean ± SD
Bougrine et al., 2024b	18.3 ± 0.5	18–19	Mean ± SD
Sánchez-Redondo et al., 2025	17 ± 1	Junior/U19 category	Mean ± SD
Bezuglov et al., 2025a	16.5	15.1–17.8	Median (IQR 1.2)

Bezuglov et al., 2025b	16.5	15.1–17.8	Median (IQR 1.2); same cohort as 2025a
Bougrine et al., 2025	16.76 ± 0.4	15–18 (eligibility)	Mean ± SD
Miraftabi et al., 2025	18.1 ± 0.9	NR	Mean ± SD
Hemmat et al., 2025	16.7 ± 0.86	NR	Mean ± SD
Amor et al., 2025	16.9 ± 0.7	16–18	Mean ± SD
Alejo et al., 2026	16.3 ± 0.5	Junior/U19 category	Mean ± SD (Methods: 16 ± 1)
Yasli et al., 2026	14.07 ± 0.26	13–15 (eligibility)	Mean ± SD

Notes: Age data are presented as reported in the original publications. Values are mean ± SD unless otherwise indicated. Adolescence was defined as 10–19 years, inclusive (i.e., ≥10 and <20 years). IQR, interquartile range; NR, not reported; SD, standard deviation; SEM, standard error of the mean. “NR” indicates that an age range or age-specific eligibility criterion was not reported and was not interpreted as confirmation that all participants were aged 10–19 years. “Age-uncertain” indicates that the reported mean or median age was within the WHO adolescent range but that an upper age limit could not be verified. Companion reports from the same cohort were counted once when summarising cohorts.

Supplementary Table 6. Safety and adverse-event findings of the included studies.

Study	Sample / sport	Caffeine dose & timing	AE assessment	Principal safety / adverse-event findings
Alejo 2026	13 trained youth cyclists	6 mg/kg, ~60 min post-race, pre-TT	Assessed & reported	All completed; no adverse events reported; HR, RPE and mood unchanged vs placebo.
Amor 2025	24 adolescent male volleyball	3 mg/kg capsule / mouth rinse; 08:00/12:00/18:00	Assessed & reported	Mild and infrequent; headache 25% with 12:00 capsule (p=0.031); tachycardia, anxiety and GI <8.3%; no sleep disturbance at 24 h; mouth rinse caused fewer effects.
Bezuglov 2025a	54 elite youth (15-17) soccer	400 mg single dose	Assessed & reported	Total AE rate not significantly raised (55.5% vs 40.7% placebo, p=0.28); headache 18.5% vs 0% (p=0.019); GI 11.1%, tachycardia 3.7%, insomnia 7.4%.
Bezuglov 2025b	54 elite youth (15-17) soccer	400 mg, 60 min pre	Load/physiologic only	No AE questionnaire; no effect on RPE, post-exercise HR, HR recovery, or 24 h muscle soreness.
Bougrine 2025	17 adolescent female handball	6 mg/kg +/- music, morning	Assessed & reported	Low incidence; same-day headache/tachycardia/GI 5.9-11.8%; 24 h insomnia 11.8%; no significant or serious effects.
Bougrine 2024a	15 low-caffeine female athletes	3 or 6 mg/kg, morning vs evening	Assessed & reported	Evening intake markedly increased effects; evening 6 mg/kg at 24 h: headache 40%, insomnia 47%, GI 27%; same-day tachycardia 33%.
Bougrine 2024b	16 adolescent female team-sport (low consumers)	3 / 6 / 9 mg/kg, 60 min pre	Assessed & reported	9 mg/kg had most effects: 24 h diuresis 31%, GI 31%, headache 25%, insomnia 25%; dose effect on GI (p=0.001); 6 mg/kg best balance.
Delleli 2023	16 male taekwondo	3 mg/kg +/- music	Assessed & reported	GI questionnaire used; moderate headache 2/16, diarrhoea 4-5/16, abdominal pain 3/16; no severe symptoms.
Abian-Vicen 2014	16 adolescent basketball	3 mg/kg caffeinated energy drink	Assessed & reported	Vs placebo: activeness/excitement 37.5% vs 0% (p<0.05); headache 6.3%, GI 12.5%, insomnia 12.5%; no palpitations or anxiety; rated mild.

Study	Sample / sport	Caffeine dose & timing	AE assessment	Principal safety / adverse-event findings
Stojanovic 2022	basketball players, AM vs PM	3 mg/kg; 10:00 vs 21:00	Assessed & reported	Evening 24 h: headache 18%, insomnia 27%; evening 30 min: diuresis 45%, tachycardia 27%, abdominal 18%; no serious events.
Spineli 2020	adolescent athletes (CYP1A2)	6 mg/kg	Assessed & reported	No side effects reported during the trial or within 24 h.
Russell 2020	academy rugby	400 mg gum (~4.1 mg/kg), half-time	Mentioned, not quantified	No GI discomfort with caffeine gum; adverse events not systematically quantified.
Guttierres 2013	20 adolescent (16.1 y) soccer	~7.2 mg/kg caffeinated sports drink	Physiologic safety noted	No symptomatic AEs; judged physiologically safe under heat; HR +~4 bpm; low diuresis-related dehydration risk.
Spineli 2024	74 adolescent male (ACE)	6 mg/kg, 1 h pre	Load/physiologic only	No symptomatic AEs reported; higher HR/RPE attributed to longer exercise time and internal load.
Scanlan 2019	21 elite basketball	3 mg/kg	Individual response only	No symptomatic AEs; 1/21 a negative performance responder (dribble deficit).
Astley 2017	young judo athletes	4 mg/kg	Load/physiologic only	No symptomatic AEs; HR unchanged; no AE questionnaire.
Gallo-Salazar 2015	14 elite junior tennis (16+/-1)	3 mg/kg caffeinated energy drink	Load/physiologic only	No AE questionnaire; sweat rate +~33.5% and greater dehydration (p=0.04); HR unaffected; nervousness/insomnia noted from prior literature.
Arazi 2016	10 adolescent female karate	2 / 5 mg/kg	Perceptual only	No symptomatic AEs; reduced RPE and pain at 5 mg/kg.
Jordan 2014	17 elite youth male soccer	6 mg/kg	Physiologic cues only	AEs not quantified; HR unchanged; some athletes described feeling hyper / heart rate up.
Pettersen 2014	19 young soccer	6 mg/kg, 65 min pre	Load/performance only	No symptomatic AEs; no effect on match activity, fatigue resistance or HR load.
Yasli 2026	soccer (practice conditions)	3 mg/kg coffee	Asked; mild/unattributed	GI discomfort queried; sour stomach in 5/56 visits, not clearly attributed to caffeine.
Hemmat 2025	20 adolescent male basketball	~3 mg/kg gum, 15 min pre	Not reported	No AE data; dose chosen for efficacy/safety balance.

Study	Sample / sport	Caffeine dose & timing	AE assessment	Principal safety / adverse-event findings
Miraftabi 2025	13 male soccer	8 x 750 mg mouth rinse	Not reported	No safety outcomes reported.
Sanchez-Redondo 2025	13 trained youth cyclists	3 mg/kg post-work, pre-TT	Not reported	No AE data; moderate dose chosen to avoid side effects in younger athletes; HR/RPE/mood unchanged.
Ghazaleh 2024	10 high-trained male (15-17) futsal	6 mg/kg, 60 min pre	Not reported	No AE quantification; no safety signal in EMG outcomes.
Ouergui 2023	taekwondo (sex/level strata)	3 mg/kg	Not reported	Only consent mention of potential effects; dose taken from prior minimal-to-no-side-effect work.
Bougrine 2023	13 adolescent female handball (Ramadan)	~6 mg/kg	Not reported (context)	No caffeine AE questionnaire; negative events were Ramadan-related sleep/performance changes.
Ellis 2019	16 elite adolescent male soccer	1 / 2 / 3 mg/kg	Not reported	No AE data; introduction notes high doses raise HR, tension, insomnia and vomiting; low doses may reduce effects.
Egesoy 2020	soccer players	6 mg/kg coffee	Not reported	No adverse-event or side-effect reporting.
Dos Santos 2023	adolescent male (ADORA2A/CYP1A2)	acute dose	Not reported	No AEs reported; introduction labels caffeine a safe ergogenic aid.
Collomp 1992	trained/untrained swimmers	250 mg	Not reported	No adverse events reported (early study).
Sasaki 1987	prolonged-running participants	~384 mg	Not reported	No adverse-event evidence.

AE, adverse event; GI, gastrointestinal; HR, heart rate; RPE, rating of perceived exertion; TT, time trial. Percentages are incidence within the caffeine condition as reported in each study; "up to" values denote the highest-risk dose-timing cell. The AE-assessment column distinguishes three categories. Actively assessed ("Assessed & reported"): a dedicated side-effect/adverse-event instrument was administered and results reported. Passively mentioned (e.g., "Load/physiologic only", "Mentioned, not quantified", "Physiologic cues only", "Perceptual only", "Asked; mild/unattributed"): symptoms or physiological signals were noted incidentally but no systematic AE instrument was used. Not assessed ("Not reported"): adverse events were not evaluated. The absence of reported events in the passively mentioned and not-assessed categories indicates incomplete monitoring, not demonstrated safety.

Supplementary Note 1. Effect-size calculation

For a crossover comparison, the uncorrected standardized mean difference was calculated as $d = (MCAF - MPLA) / SDpooled$, where $SDpooled = \sqrt{(SDCAF^2 + SDPLA^2) / 2}$. The small-sample correction was $J = 1 - 3 / (4df - 1)$, and Hedges' g was calculated as $g = J \times d$.

Supplementary Table 7. Small-study effect (Egger-type) regression diagnostics by outcome domain.

**Outcome domain**	**Independent clusters (k)**	**Egger-type slope**	**SE**	**p**	**Small-study asymmetry**
Physical performance	24	7.99	1.99	< 0.001	Significant
All domains combined	32	8.17	—	0.015	Significant
Sport-specific performance	11	7.23	—	0.299	Not significant
Perceptual response	13	4.95	—	0.144	Not significant
Physiological response	10	−1.90	—	0.479	Not significant
Cognitive performance	2	—	—	—	Not evaluated (only 2 clusters)
Psychological response	2	—	—	—	Not evaluated (only 2 clusters)

Note: Small-study effects were screened using an Egger-type regression of each effect size on its standard error, fitted within the correlated-and-hierarchical-effects (CHE) model with cluster-robust (CR2) variance estimation (see Section 3.6). The test was interpreted only for domains with at least ten independent study clusters; cognitive and psychological response each rested on only two clusters and could not be evaluated. Given the small number of studies, effect-size dependence, and the predominance of small studies, the power of the asymmetry test was limited and significant slopes should be read as suggestive rather than confirmatory. SE, standard error.
